# AMEERA-1 phase 1/2 study of amcenestrant, SAR439859, in postmenopausal women with ER-positive/HER2-negative advanced breast cancer

**DOI:** 10.1038/s41467-022-31668-8

**Published:** 2022-07-15

**Authors:** Aditya Bardia, Sarat Chandarlapaty, Hannah M. Linden, Gary A. Ulaner, Alice Gosselin, Sylvaine Cartot-Cotton, Patrick Cohen, Séverine Doroumian, Gautier Paux, Marina Celanovic, Vasiliki Pelekanou, Jeffrey E. Ming, Nils Ternès, Monsif Bouaboula, Joon Sang Lee, Anne-Laure Bauchet, Mario Campone

**Affiliations:** 1 Massachusetts General Hospital Cancer Center, Harvard Medical School, Boston, MA USA; 2 Memorial Sloan Kettering Cancer Center, New York, NY USA; 3 University of Washington Medical Center, Seattle Cancer Care Alliance, Seattle, WA USA; 4 Hoag Family Cancer Institute, Newport Beach, CA USA; 5 University of Southern California, Los Angeles, CA USA; 6 Sanofi, Paris, France; 7 Sanofi, Montpellier, France; 8 Sanofi, Cambridge, MA USA; 9 Sanofi, Bridgewater, NJ USA; 10 Institut de Cancérologie de l’Ouest, René Gauducheau, Saint-Herblain, France; 11 Present Address: Bayer US-Pharmaceuticals, Cambridge, MA USA

**Keywords:** Breast cancer, Cancer genomics, Cancer therapeutic resistance

## Abstract

AMEERA-1 is a Phase 1/2 open-label single-arm study evaluating once-daily (QD) amcenestrant, an orally bioavailable selective estrogen receptor (ER) degrader, in postmenopausal women with ER+/HER2− advanced breast cancer (NCT03284957), who were mostly heavily pretreated (including targeted therapies and fulvestrant). In the dose escalation phase (Part A: *n* = 16), patients received amcenestrant 20-600 mg QD. Based on absence of dose-limiting toxicities, paired functional ^18^F-fluoroestradiol positron emission tomography, and pharmacokinetics, 400 mg QD was selected as recommended Phase 2 dose (RP2D) for the dose expansion phase (Part B: *n* = 49). No Grade ≥3 treatment-related adverse events or clinically significant cardiac/eye toxicities were reported. The Part B primary endpoint, confirmed objective response rate (ORR) was 3/45 at the interim analysis and 5/46 (10.9%) at the final analysis. The overall clinical benefit rate (CBR) was 13/46 (28.3%). CBRs among patients with baseline wild-type and mutated *ESR1* were 9/26 (34.6%) and 4/19 (21.1%), respectively. Paired tumor biopsy and cell-free DNA analyses revealed ER inhibition and degradation, and a reduction in detectable *ESR1* mutations, including *Y537S*. In conclusion, amcenestrant at RP2D of 400 mg QD for monotherapy is well-tolerated with no dose-limiting toxicities, and demonstrates preliminary antitumor activity irrespective of baseline *ESR1* mutation status.

## Abstract (web summary)

There is a need for potent and non-toxic estrogen receptor (ER) antagonists to overcome the limitations of existing endocrine therapies. Here the authors report the results from Arm 1 of the Phase 1/2 study (AMEERA-1) among postmenopausal women with ER+/HER2− advanced breast cancer, which evaluates the safety, antitumor activity, pharmacokinetics, and pharmacodynamics of amcenestrant administered as monotherapy.

## Introduction

Among women worldwide, breast cancer is the most prevalent form of cancer, accounting for 24.2% of all cancer diagnoses and 15.0% of cancer-related mortality^1^. The majority (68–73%) of women with breast cancer present with hormone receptor-positive (HR+; estrogen receptor-positive [ER+] and/or progesterone receptor-positive [PgR+]) and human epidermal growth factor receptor 2-negative (HER2−) disease, with 14–15% presenting with HER2+ disease and 10–12% with triple-negative disease^2,3^.

Endocrine therapies, including aromatase inhibitors (AIs, e.g., letrozole), selective ER modulators (SERMs, e.g., tamoxifen), and selective ER degraders (SERDs, e.g., fulvestrant) that block ER signaling via ER inhibition, modulation, or degradation, and hence estrogen-promoted tumor growth, are mainstays of treatment for patients with HR+/HER2− breast cancer^4^.

Although 5-year relative survival rates in the US among women with HR+/HER2− breast cancer are high with localized (100%) or regional (90%) disease^5^, approximately 30% of patients will relapse, often with metastatic disease^4,6^, which is associated with a low 5-year relative survival (30%)^5^.

Among women with advanced or metastatic disease, resistance to endocrine therapies commonly occurs, either because of the development of *ESR1* mutations in 12–39% of tumors exposed to AIs^7–10^ or poor drug bioavailability in tumors treated with fulvestrant^11^. These and other mechanisms of resistance to endocrine therapies in the metastatic setting, including overexpression of ER coactivators and loss of ER dependence via activation of other oncogenic signals such as via NF1^12–14^ or ARID1A^15,16^ loss, may differentially affect the efficacy of endocrine therapies^6^. Tumors that have developed resistance to one endocrine therapy and continue to express ER but have not lost ER dependence often respond to an alternative endocrine therapy^17^ or to a cyclin-dependent kinase (CDK) 4/6 inhibitor, which are downstream effectors^18^ and usually given in combination with endocrine therapy for synergy. For example, *ESR1* mutations detected after AI therapy for metastatic breast cancer promote AI resistance while retaining sensitivity to ER antagonism via SERMs or SERDs^8,19,20^. However, the in vivo activity of SERMs and SERDs is often compromised by properties of these drugs related to (i) partial agonism of some SERMs^21^, (ii) toxicity profiles of certain drugs potentially contributing to discontinuation^22–24^, and (iii) poor bioavailability of some compounds compromising target inhibition^25^. The latter represents a major limitation of the only approved SERD, fulvestrant^26^, whose poor oral bioavailability (requiring intramuscular injections^27^ and 1 month to reach steady state^28,29^) and pharmacodynamic limitations (the maximum 500 mg dose does not fully occupy the ER^25^) have contributed to therapy failure^30^. This represents a particular problem for the *Y537S* mutation in *ESR1*, which is the second most common *ESR1* mutation (14%) requiring higher doses of the drug to achieve occupancy and inhibition of the ER ligand-binding domain and is associated with fulvestrant resistance in vivo^20^. Thus, there remains a need for potent and non-toxic pure ER antagonists to overcome the limitations of existing endocrine therapies.

Amcenestrant (SAR439859) is an orally bioavailable SERD that demonstrates pure ER antagonism in vivo^31^. Compared with other SERD agents, amcenestrant provides optimal ER antagonism and degradation. Amcenestrant robustly inhibits the ER signaling pathway in multiple ER+ breast cancer cell lines, including fulvestrant- and tamoxifen-resistant lines, as well as cell lines harboring ER mutations^32–34^. Preclinical data show that amcenestrant degrades the ER with high efficacy (98%) and potency (0.2 nM) comparable to fulvestrant in vitro activity, is metabolically stable, and has no off-target activity (half maximal inhibitory concentration ≤1 µM) in a large in vitro safety screen panel^33^. Moreover, in a mouse model of subcutaneous *MCF7-Y537S* mutant ERα + breast cancer, ^18^F-fluoroestradiol positron emission tomography (^18^F-FES PET) imaging confirmed that amcenestrant dose-dependently inhibited tumor uptake of ^18^F-FES, which correlated with immunohistochemical scoring for ERα expression, and ^18^F-fluorothymidine (FLT) PET showed a significant decrease in tumoral FLT accumulation when amcenestrant was combined with palbociclib^34^. Taken together, these findings support the further development of amcenestrant, alone and in combination therapy, for the treatment of ER+ breast cancer.

Here, we report the final results from Arm 1 of the Phase 1/2 study (AMEERA-1) among postmenopausal women with ER+/HER2− advanced breast cancer, which evaluates the safety, antitumor activity, pharmacokinetics, and pharmacodynamics of amcenestrant administered as monotherapy in dose escalation (Part A) and dose expansion (Part B).

## Results

The first patient was enrolled on 6 November 2017 and the last patient was enrolled on 26 March 2020. Each patient had data collected until the end of treatment, which was 22–30 days after the last administration of study treatment. All results are reported as of the cutoff date of 30 March 2021.

### Part A dose-escalation phase: patients

**Fig. 1:**
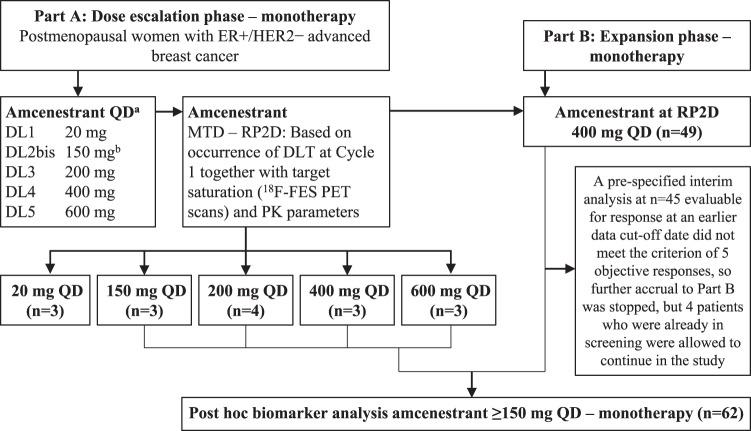
Study flow diagram for Parts A and B. During Part A, patients were enrolled into a dose escalation phase of amcenestrant monotherapy from 20-600 mg once daily. During Part B, patients were enrolled into a dose-expansion phase at the RP2D determined in Part A. ^a^The first patient treated at each new DL will be followed for a minimum of 1 week prior to enrolling and treating two additional patients. If none of the three patients experience a DLT, the next cohort starts one DL higher. If one of the three patients experiences a DLT, up to three additional patients are treated at this DL. If two or more patients experience a DLT, the maximum administered dose is reached. ^b^DL2bis is investigated if at least one patient at DL1 shows ≤ 30% inhibition of the target by ^18^F-FES PET or all patients treated at DL2 have >85% inhibition of the target by ^18^F-FES PET. DL dose level, DLT dose-limiting toxicity, ER+ estrogen receptor positive, ^18^F-FES PET ^18^F-fluoroestradiol positron emission tomography, HER2− human epidermal growth factor receptor 2-negative, MTD maximum tolerated dose, RP2D recommended Phase 2 dose, PK pharmacokinetics, QD once daily.

In dose escalation (Part A), a total of 16 patients were treated at five dose levels of amcenestrant once daily (QD) monotherapy with dose escalation proceeding according to criteria described in Fig. 1 and Table 1: 20 mg (*n* = 3), 150 mg (*n* = 3), 200 mg (*n* = 4), 400 mg (*n* = 3), and 600 mg (*n* = 3). Among these patients, the median age was 59.5 (range 40–79) years, the Eastern Cooperative Oncology Group (ECOG) performance score (PS) was 0 in 62.5% or 1 in 37.5%, 93.8% had visceral metastases (Table 2). The majority of patients were heavily pretreated: the number of prior lines of therapy in the advanced setting ranged from 1 to 8 (93.8% had received ≥2 prior lines); all had received prior endocrine therapy; 81.3% had received prior targeted therapy; and 56.5% prior fulvestrant (Table 2).

**Table 1:** Pre-specified dose-escalation schedule for amcenestrant monotherapy in Part A.

Dose level (DL)^a^	Amcenestrant dose (mg)
DL(−1) QD	10 once daily
DL1 QD	20 once daily
DL1bis QD	50 once daily
DL2 QD	100 once daily
DL2bis QD	150 once daily
DL3 QD	200 once daily
DL4 QD	400 once daily
DL4bis BID	200 twice daily
DL5 QD	600 once daily
DL5bis BID	300 twice daily

A BID schedule of administration may be added during the study; the starting dose will be a DL of the same dose intensity as the highest cleared DL with QD schedule. Other schedules of administration may be added during the study.

*BID* twice daily, *QD* once daily.

^a^Additional intermediate or higher dose levels can be tested after the agreement between Sponsor and Investigators (study committee).

**Table 2:** Demographic and patient characteristics at baseline in Parts A and B (safety populations) and the biomarker population (Parts A and B excluding the amcenestrant QD 20-mg dose).

	Amcenestrant dose escalation Part A (*N* = 16)	Amcenestrant 400 mg Part B (*N* = 49)	Amcenestrant ≥150 mg Biomarkers (*N* = 62)
Age, years, median (range)	59.5 (40–79)	63.0 (37–88)	63.0 (37–88)
Race, *n* (%)			
White	10/10 (100)	35/36 (97.2)	44/45 (97.8)
Asian	0	1/36 (2.8)	1/45 (2.2)
Missing^a^	6	13	17
ECOG PS, *n* (%)			
0	10 (62.5)	29 (59.2)	37 (59.7)
1	6 (37.5)	20 (40.8)	25 (40.3)
*ESR1* status at baseline, *n* (%)			
Wild-type	5 (31.3)	27 (55.1)	31 (50.0)
Mutated	11 (68.8)	21 (42.9)	30 (48.4)
Missing	0	1 (2.0)	1 (1.6)
Visceral metastases, *n* (%)	15 (93.8)	46 (93.9)	58 (93.5)
Prior anti-cancer treatment for advanced or metastatic disease, *n* (%)			
Prior chemotherapy	8 (50.0)	20 (40.8)	26 (41.9)
Anthracyclines	2 (12.5)	2 (4.1)	3 (4.8)
Taxanes	4 (25.0)	7 (14.3)	10 (16.1)
Other	7 (43.8)	15 (30.6)	20 (32.3)
Prior hormone therapy	16 (100)	49 (100)	62 (100)
Aromatase inhibitors	15 (93.8)	47 (95.9)	59 (95.2)
SERD (fulvestrant)	9 (56.3)	22 (44.9)	29 (46.8)
SERM	5 (31.3)	14 (28.6)	18 (29.0)
Prior targeted therapy	13 (81.3)	35 (71.4)	45 (72.6)
CDK4/6 inhibitors	12 (75.0)	30 (61.2)	39 (62.9)
mTOR inhibitors	7 (43.8)	16 (32.7)	21 (33.9)
PI3K inhibitors	0	6 (12.2)	6 (9.7)
Angiogenesis inhibitors	0	1 (2.0)	1 (1.6)
Prior PD-1 inhibitor	1 (6.3)	0	1 (1.6)
Other	1 (6.3)	1 (2.0)	2 (3.2)
Prior lines of therapy in advanced settings			
Minimum–maximum	1–8	1–6	1–8
By category, *n* (%)			
1 line	1 (6.3)	14 (28.6)	15 (24.2)
2 lines	7 (43.8)	11 (22.4)	17 (27.4)
≥3 lines	8 (50.0)	24 (49.0)	30 (48.4)
Prior lines of hormone therapy in advanced settings			
Minimum–maximum	1–6	1–4	1–6
By category, *n* (%)			
1 line	3 (18.8)	17 (34.7)	20 (32.3)
2 lines	7 (43.8)	15 (30.6)	21 (33.9)
≥3 lines	6 (37.5)	17 (34.7)	21 (33.9)
Time from first diagnosis to first study treatment administration, years, median (range)	9.7 (2.3–22.7)	6.0 (0.8–24.3)	6.7 (0.8–24.3)

*CDK 4/6* cyclin-dependent kinase 4 and 6, *ECOG PS* Eastern Cooperative Oncology Group performance score, *ESR1* estrogen receptor 1, *PD-1* programmed cell death protein 1, *PI3K* phosphoinositide 3-kinase, *SERD* selective estrogen receptor degrader, *SERM* selective estrogen receptor modulator.

^a^Data on race were not collected for French sites.

### Part A dose escalation phase: treatment exposure

The median duration of treatment was 23.6 weeks (range 4–90 weeks). Median (min–max) relative dose intensity was 100% (86–100%). One (6.3%) patient had a dose reduction to 200 mg at the 400 mg dose level. All 16 patients discontinued the study treatment because of progressive disease.

Although six patients were recruited as planned for the BID regimen, only two patients were DLT-evaluable, including two patients who were unable to have ^18^F-FES PET scans because sites were unable to perform them due to the coronavirus pandemic. In addition, four patients had early progressive disease occurring before the end of cycle 2. Thus, it was decided not to further pursue exploration of this dosing regimen. A summary of these results is provided in Supplementary Notes: BID dosing regimen.

### Part A dose-escalation phase: primary endpoints

No dose-limiting toxicity (DLT) was observed during the DLT observation period (cycle 1) at any amcenestrant dose, no adverse event (AE) met DLT criteria definition in subsequent cycles, and the MTD was not reached. The determination of the recommended Phase 2 dose (RP2D) is described in the Part A pharmacodynamics section.

### Part A dose-escalation phase: secondary endpoints: safety

The most frequently reported (occurring in at least three patients) treatment-emergent adverse events (TEAEs) specifically related to amcenestrant were as follows: hot flush (*n* = 5; 31.3%), diarrhea and nausea (*n* = 4 each; 25.0%), as well as decreased appetite, constipation, night sweats, and asthenia (*n* = 3 each; 18.8%) (Table 3); of these, most were Grade 1. No TEAE led to treatment discontinuation.

**Table 3:** Overview of adverse event profile in Part A and Part B (patients with all TEAEs occurring in at least three patients and their associated treatment-related TEAEs)—safety populations.

	TEAEs, *n* (%)				Treatment-related TEAEs, *n* (%)			
	All grades	Grade 1	Grade 2	Grade ≥3	All Grades	Grade 1	Grade 2	Grade ≥3
*Part A (N* *=* *16)*								
Any TEAE	16 (100)	5 (31.3)	7 (43.8)	4 (25.0)	13 (81.3)	10 (62.5)	3 (18.8)	0
Constipation	6 (37.5)	5 (31.3)	1 (6.3)	0	3 (18.8)	3 (18.8)	0	0
Decreased appetite	6 (37.5)	6 (37.5)	0	0	3 (18.8)	3 (18.8)	0	0
Diarrhea	6 (37.5)	5 (31.3)	1 (6.3)	0	4 (25.0)	3 (18.8)	1 (6.3)	0
Hot flush	6 (37.5)	6 (37.5)	0	0	5 (31.3)	5 (31.3)	0	0
Nausea	6 (37.5)	3 (18.8)	3 (18.8)	0	4 (25.0)	3 (18.8)	1 (6.3)	0
Arthralgia	5 (31.3)	2 (12.5)	3 (18.8)	0	2 (12.5)	1 (6.3)	1 (6.3)	0
Asthenia	4 (25.0)	2 (12.5)	2 (12.5)	0	3 (18.8)	3 (18.8)	0	0
Dyspnea	4 (25.0)	2 (12.5)	1 (6.3)	1 (6.3)	1 (6.3)	1 (6.3)	0	0
Fatigue	4 (25.0)	3 (18.8)	0	1 (6.3)	2 (12.5)	2 (12.5)	0	0
Urinary tract infection	4 (25.0)	0	3 (18.8)	1 (6.3)	0	0	0	0
Back pain	3 (18.8)	2 (12.5)	0	1 (6.3)	1 (6.3)	1 (6.3)	0	0
Hypoesthesia	3 (18.8)	3 (18.8)	0	0	0	0	0	0
Night sweat	3 (18.8)	3 (18.8)	0	0	3 (18.8)	3 (18.8)	0	0
*Part B (N* *=* *49)*								
Any TEAE	49 (100)	11 (22.4)	22 (44.9)	16 (32.7)	26 (53.1)	21 (42.9)	5 (10.2)	0
Constipation	14 (28.6)	12 (24.5)	2 (4.1)	0	3 (6.1)	2 (4.1)	1 (2.0)	0
Vomiting	14 (28.6)	10 (20.4)	3 (6.1)	1 (2.0)	4 (8.2)	4 (8.2)	0	0
Fatigue	12 (24.5)	8 (16.3)	2 (4.1)	2 (4.1)	2 (4.1)	2 (4.1)	0	0
Abdominal pain	11 (22.4)	8 (16.3)	3 (6.1)	0	2 (4.1)	2 (4.1)	0	0
Nausea	11 (22.4)	9 (18.4)	1 (2.0)	1 (2.0)	2 (4.1)	2 (4.1)	0	0
Arthralgia	10 (20.4)	7 (14.3)	3 (6.1)	0	4 (8.2)	3 (6.1)	1 (2.0)	0
Asthenia	10 (20.4)	7 (14.3)	3 (6.1)	0	2 (4.1)	2 (4.1)	0	0
Diarrhea	8 (16.3)	5 (10.2)	2 (4.1)	1 (2.0)	1 (2.0)	1 (2.0)	0	0
Dyspnea	8 (16.3)	6 (12.2)	2 (4.1)	0	1 (2.0)	1 (2.0)	0	0
Decreased appetite	7 (14.3)	5 (10.2)	2 (4.1)	0	2 (4.1)	2 (4.1)	0	0
Hot flush	7 (14.3)	5 (10.2)	2 (4.1)	0	5 (10.2)	5 (10.2)	0	0
Abdominal pain upper	6 (12.2)	2 (4.1)	4 (8.2)	0	1 (2.0)	1 (2.0)	0	0
Back pain	5 (10.2)	3 (6.1)	2 (4.1)	0	0	0	0	0
Urinary tract infection	5 (10.2)	0	5 (10.2)	0	1 (2.0)	0	1 (2.0)	0
Cough	4 (8.2)	4 (8.2)	0	0	1 (2.0)	1 (2.0)	0	0
Gastroesophageal reflux disease	4 (8.2)	4 (8.2)	0	0	3 (6.1)	3 (6.1)	0	0
Hypertension	4 (8.2)	0	4 (8.2)	0	1 (2.0)	0	1 (2.0)	0
Pyrexia	4 (8.2)	3 (6.1)	1 (2.0)	0	0	0	0	0
ALT increased	3 (6.1)	0	0	3 (6.1)	0	0	0	0
AST increased	3 (6.1)	0	0	3 (6.1)	0	0	0	0
Depression	3 (6.1)	3 (6.1)	0	0	2 (4.1)	2 (4.1)	0	0
Dry skin	3 (6.1)	2 (4.1)	1 (2.0)	0	1 (2.0)	1 (2.0)	0	0
Edema peripheral	3 (6.1)	3 (6.1)	0	0	1 (2.0)	1 (2.0)	0	0
Headache	3 (6.1)	3 (6.1)	0	0	0	0	0	0
Nasopharyngitis	3 (6.1)	3 (6.1)	0	0	0	0	0	0
Pain in extremity	3 (6.1)	2 (4.1)	1 (2.0)		0	0	0	0
Paresthesia	3 (6.1)	3 (6.1)	0	0	1 (2.0)	1 (2.0)	0	0
Rhinitis allergic	3 (6.1)	2 (4.1)	1 (2.0)	0	0	0	0	0

*ALT* alanine aminotransferase, *AST* aspartate aminotransferase, *TEAE* treatment-emergent adverse event.

Cardiac non-treatment-related TEAEs were reported in 2 patients (Grade 1 palpitations and Grade 2 prolonged QT interval). An eye disorder treatment-related TEAE was reported in 1 patient (Grade 1 photophobia) with an additional two eye disorder non-treatment-related TEAEs in another patient (Grade 1 photophobia and eye irritation).

Serious TEAEs associated with disease progression were reported in three patients and were considered unrelated to amcenestrant (Grade 3 back pain in a patient treated at 150 mg QD who later died due to disease progression 22 days after amcenestrant discontinuation; Grade 3 dyspnea in one patient treated at 200 mg QD; and Grade 3 fatigue in one patient treated at 600 mg QD). Three other patients enrolled in Part A died (two due to disease progression 123 and 189 days after amcenestrant discontinuation and one due to an unknown reason 44 days after amcenestrant discontinuation).

### Part A dose-escalation phase: secondary endpoints: pharmacokinetics

**Fig. 2:**
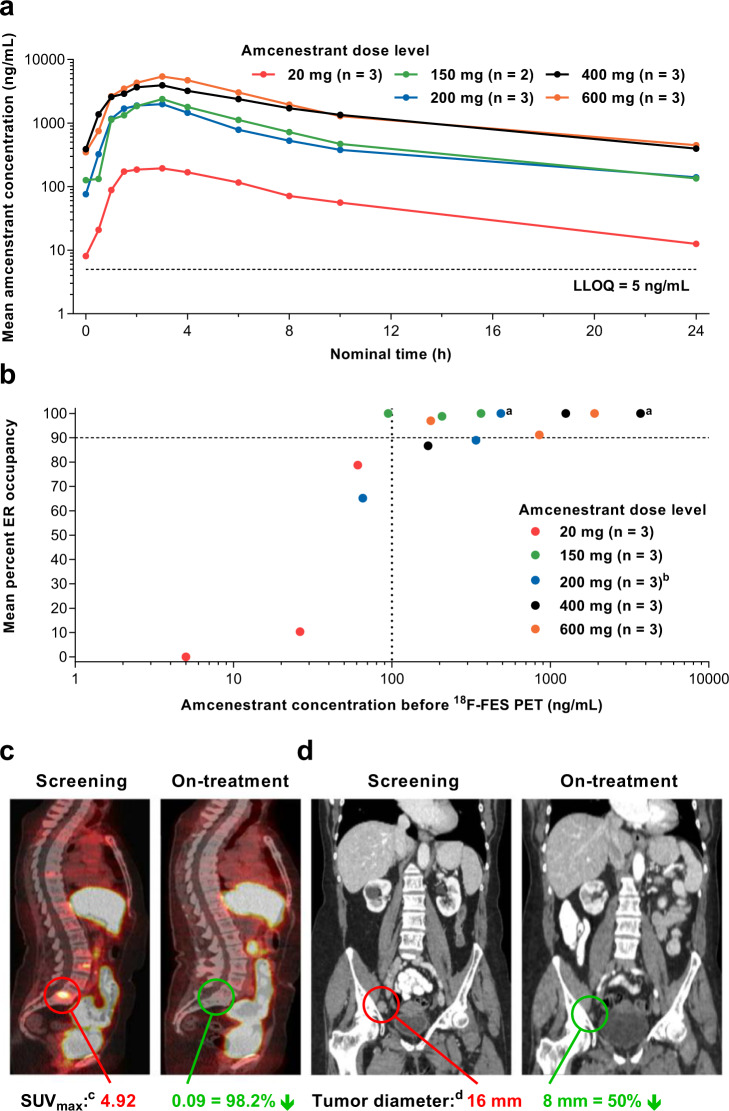
Part A dose escalation: amcenestrant plasma concentration-time profiles, pharmacokinetic/pharmacodynamic relationships, and ^18^F-FES PET/CT images. **a** Mean amcenestrant plasma concentration-time profiles observed following repeated oral administration (cycle 1, day 22) under fasting conditions; **b** pharmacokinetic/pharmacodynamic relationship between plasma concentrations of amcenestrant measured just before ^18^F-FES radioisotope administration and individual mean percent occupancy of estrogen receptors (mean percent reduction in ^18^F-FES SUV_max(cor)_); **c** representative ^18^F-FES PET; and **d** coronal CT images at screening and on-treatment with amcenestrant 150 mg. ^18^F-FES PET/CT scans were performed 16–24 h after the previous amcenestrant dose, except for two patients whose scans occurred 8 h after the previous dose^a^. Of 16 patients, 14 patients had scans at baseline and between 11–15 days after first amcenestrant administration (as per protocol) and two patients had post-baseline scans on days 10 and 28, respectively. The patient with a post-baseline scan on day 10 was included in the pharmacodynamic analysis because the scan occurred after ≥8 days of continuous treatment, but the patient receiving amcenestrant 200 mg with a post-baseline scan on day 28 was excluded from pharmacodynamic analysis and dose escalation decisions^b^. ^c^Annotated example of the reduction in SUV_max(cor)_ for the first index lesion in the 5th lumbar vertebra; physiologic ^18^F-FES avidity is noted in the liver, intestines, and bladder. ^d^Annotated example of the reduction in tumor diameter of a right pelvic lymph node in the associated CT scan. CT computerized tomography, ER estrogen receptor ^18^F-FES PET ^18^F-fluoroestradiol positron emission tomography, LLOQ lower limit of quantification, SUV_max(cor)_ maximum standardized uptake value, standardized by body weight and corrected for background.

Pharmacokinetic parameters obtained after single and repeated amcenestrant administrations are presented in Supplementary Tables S1 and S2, respectively. Following a single dose on day 1, the pharmacokinetic concentration-time profile obtained from a total of 16 patients in dose escalation generally showed a moderate absorption rate starting from the 150-mg dose (median time to maximum concentration [*t*_max_] ≈ 3 h) followed by a biphasic elimination profile. After repeated dosing (day 22), median *t*_max_ generally remained around 3 h (Fig. 2a). No accumulation was observed on day 22 with amcenestrant at doses ≥400 mg QD (Supplementary Table S3). Exposure increased with dose and did not deviate significantly from dose proportionality between amcenestrant 20–600 mg QD after single or multiple administrations (Supplementary Table S4).

Food intake (moderate fat breakfast) modestly increased exposure (Supplementary Table S5) and median *t*_max_ was delayed by ≈1 h. However, this effect was not considered as clinically relevant compared with the overall variability after a single-dose administration.

The 4β-hydroxycholesterol pre-/post-treatment geometric mean ratio, measured after 4 weeks of repeated oral administration of amcenestrant, was consistently above 1, starting from the 200-mg QD dose. This ratio indicated that ≥200 mg QD of amcenestrant can induce cytochrome P450 (CYP) 3 A liver enzyme activity and that this induction effect was higher with increasing daily dose intensity (Supplementary Table S6).

### Part A dose-escalation phase: secondary endpoints: pharmacodynamics

Functional pharmacodynamics was assessed using ^18^F-FES PET/computerized tomography (CT) imaging. Because ^18^F-FES PET/CT scan results for patients treated at the 20-mg dose level did not meet the criteria for testing the 50-mg and 100-mg doses (Fig. 2b and Supplementary Methods: Dose escalation amcenestrant monotherapy), the next dose level tested was 150 mg. High ER occupancy levels were observed from this dose level (Fig. 2b).

A strong pharmacokinetic/pharmacodynamic relationship was established between amcenestrant plasma concentrations and ^18^F-FES PET percent occupancy of the ER, with an amcenestrant concentration of 100 ng/mL associated with near 90% occupancy or more (Fig. 2b). As per protocol, because all patients treated at the 150-mg dose showed >90% ER occupancy (Fig. 2b) and no DLTs occurred at any dose without reaching the MTD, amcenestrant 400 mg QD was chosen as the RP2D for the dose-expansion phase. Thus, amcenestrant met its primary endpoints for Part A. Median (range) occupancy was 100% (87–100%) with amcenestrant 400 mg QD and 97% (0–100%) across all dose levels. In addition, ^18^F-FES avidity in pretreatment scans was detected at multiple tumor sites, including lymph nodes and bone, which was markedly reduced in on-treatment scans (Fig. 2c).

### Part A dose-escalation phase: secondary endpoints: antitumor activity

**Fig. 3:**
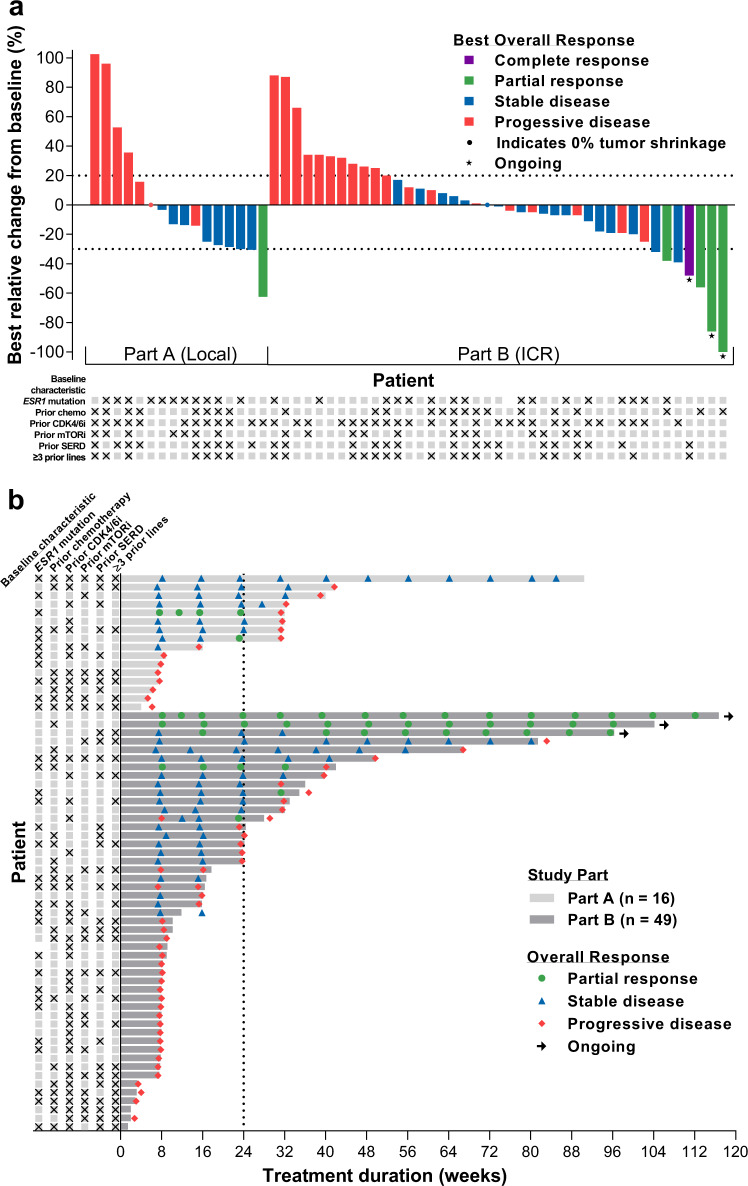
Waterfall and swimmer plots. **a** Waterfall plot of best relative change from baseline in the sum of diameters of target lesions in the response-evaluable populations of Part A by local investigators/radiologists review (*n* = 16) and Part B by independent central review (*n* = 41; three patients were missing relative change data and two patients had a non-evaluable best overall response) and **b** Swimmer plot of duration of treatment in the safety populations of Part A (*n* = 16) and Part B (*n* = 49) with overall responses assessed by local investigators/radiologists review. CDK4/6i cyclin-dependent kinase 4/6 inhibitor, chemo chemotherapy, ICR independent central review, Local local investigators/radiologists review, mTORi mammalian target of rapamycin inhibitor, SERD selective estrogen receptor degrader. Checkboxes correspond to baseline characteristics.

All 16 patients were evaluable as per Response Evaluation Criteria In Solid Tumors (RECIST) v.1.1 per investigator/local radiologists review, 1 (6.3%) patient, treated at 150 mg QD, had a confirmed partial response (PR) as best overall response, 8 (50%) patients had stable disease (SD), and 7 (43.8%) patients had progressive disease (PD), giving an objective response rate (ORR) of 6.3% and clinical benefit rate (CBR) of 50.0% (Table 3). Tumor shrinkage (decrease from baseline in the sum of diameters of target lesions) was observed in 10 (62.5%) patients (Fig. 3a).

### Part B dose-expansion phase: Patients

In dose expansion (Part B), an interim analysis was planned after 45 patients were evaluable for response, the results of which stopped accrual to Part B. By the time the 45th patient was enrolled and treated in the study, four patients were already in screening. These four additional patients were allowed to enter the trial, giving a total of 49 patients (Fig. 1) who were treated with amcenestrant 400 mg QD monotherapy, either in fasting or fed state. Among these 49 patients, median age was 63.0 (range 37–88) years, 59.2% had ECOG PS of 0, 93.9% had visceral metastases (Table 2). The majority of patients were heavily pretreated: the number of prior lines of therapy in the advanced setting ranged from 1 to 6 (71.4% had received ≥2 prior lines), all had received prior endocrine therapy, 71.4% had received prior targeted therapy, and 44.9% prior fulvestrant (Table 2).

### Part B dose-expansion phase: treatment exposure

The median duration of treatment was 10.1 weeks (range 1–117 weeks). Median (min–max) relative dose intensity was 100% (66–105%). Five (10.2%) patients had dose reductions to 200 mg QD and 2 (4.1%) patients had at least 7 consecutive days of dose omission. A total of 46 (93.9%) patients discontinued amcenestrant, including 43 (87.8%) due to progressive disease, 1 (2%) for a non-treatment-related TEAE (pneumonia), and 2 (4.1%) for other reasons (HER2 amplification detected, chest wall resection). Three (6.1%) patients remained in treatment at the date of data cutoff.

### Part B dose-expansion phase: primary endpoint

At the interim analysis of antitumor activity, which followed the Simon 2-stage design, 45 (91.8%) participants in Part B were assessed for an interim futility analysis at the data cutoff of 30 May 2020. At that time, an objective response based on independent central review (ICR) was observed in 3/45 (6.7%) participants, which did not meet the prespecified criterion of at least five responders; therefore, the primary endpoint for Part B was not met and accrual to Part B was stopped, excepting four patients who were already in screening and were allowed to continue in the study (Fig. 1). Nevertheless, ongoing participants continued to be monitored for antitumor activity, and, at the cutoff date of this final analysis, 30 March 2021, an objective response based on ICR was observed in 5 of those original 45 participants included in the interim analysis (ORR, 11.1%) (Table 4).

**Table 4:** Prespecified analyses of antitumor activity in the response-evaluable populations of Parts A and B.

Population	Part A	Part B	Part B	Part B	
Review method	Local	ICR	ICR	ICR	
Endpoint	Secondary	Interim	Primary	Secondary by baseline *ESR1* status^d^	
Subgroup				Wild-type	Mutated
Best overall response, *n* (%)					
Number	16	45	46	26	19
Complete response (CR)^a^	0	1 (2.2)	1 (2.2)	1 (3.8)	0
Partial response (PR)^a^	1 (6.3)	4 (8.9)	4 (8.7)	3 (11.5)	1 (5.3)
Stable disease (SD)	8 (50.0)	19 (42.2)	19 (41.3)	10 (38.5)	9 (47.4)
Progressive disease (PD)	7 (43.8)	19 (42.2)	20 (43.5)	11 (42.3)	8 (42.1)
Not evaluable (NE)	0	2 (4.4)	2 (4.3)	1 (3.8)	1 (5.3)
Objective response rate, *n* (%) [90% CI]^a,b^	1 (6.3) [0.3–26.4]	5 (11.1) [4.5–22.0]	5 (10.9) [4.4–21.5]	4 (15.4) [5.4–31.8]	1 (5.3) [0.3–22.6]
Clinical benefit rate, *n* (%) [90% CI]^b,c^	8 (50.0) [27.9–72.1]	13 (28.9) [18.0–42.0]	13 (28.3) [17.6–41.1]	9 (34.6) [19.4–52.6]	4 (21.1) [7.5–41.9]

*CI* confidence interval, *ESR1* estrogen receptor 1, *ICR* independent central review, *Local* investigator/local radiologist review.

^a^Confirmation of complete and partial responses are required (i.e., a second examination done ≥28 days apart, in order to confirm the antitumor response).

^b^90% CI estimated by the Clopper-Pearson interval.

^c^Clinical benefit rate: complete or partial responses or prolonged stable disease (i.e., for ≥24 weeks).

^d^One patient had missing baseline *ESR1* status.

A total of 46/49 (93.9%) patients were evaluable as per RECISTv.1.1 in Part B dose expansion. The ORR by ICR was 5/46 (10.9%), comprising 1 confirmed complete response (CR) and 4 confirmed PRs (Table 4).

### Part B dose-expansion phase: secondary endpoints: safety

All patients experienced at least one TEAE (all grades) during Part B, regardless of the relationship with amcenestrant, and one Grade ≥3 TEAE of pneumonia led to treatment discontinuation. The most frequently reported TEAEs (in at least three patients) specifically related to amcenestrant were as follows: hot flush (10.2%), vomiting and arthralgia (8.2%), and constipation and gastroesophageal reflux disease (6.1%) (Table 3); of these, most were Grade 1. At least one Grade ≥3 TEAE occurred in 16/49 (32.7%) patients; however, none of these events were considered related to amcenestrant (Table 3).

One cardiac non-treatment-related TEAE was reported (Grade 1 sinus bradycardia). Three eye disorder non-treatment-related TEAEs were reported in two patients (Grade 2 eyelid erythema and Grade 1 visual impairment in one patient, and Grade 1 macular edema in the other patient).

Serious TEAEs occurred in 13/49 (26.5%) patients (Supplementary Table S7) and none were considered related to amcenestrant by the investigator. Of the 13 patients with serious AEs, two died at 1 and 4 days after amcenestrant discontinuation. The first patient died due to pneumonia (clinical context: on amcenestrant from days 1–10, disease progression observed from day 1, Grade 3 TEAE of pneumonia on day 5, despite treatment for pneumonia the patient died on day 11), and the second due to an unknown cause (clinical context: on amcenestrant from days 1 to 64, pleural target lesion and bone non-target lesion found on CT on day 11, pleural and bone lesions had progressed and a new liver lesion was found on CT on day 53, amcenestrant was discontinued due to disease progression on day 64, the patient died on day 68). Two other patients enrolled in Part B died during the follow-up period at 32 and 69 days after amcenestrant discontinuation, both due to disease progression.

### Part B dose-expansion phase: secondary endpoints: pharmacokinetics

Pharmacokinetic parameters obtained after single and repeated QD administrations of amcenestrant 400 mg are presented in Supplementary Table S8 and were consistent with those measured during Part A. No drug accumulation was observed between day 1 and day 22 with geometric accumulation ratios of 0.93 (90% confidence interval [CI]: 0.75–1.15) and 0.81 (90% CI: 0.64–1.01) based upon the area under the curve over 24 h (AUC_0-24h_) and maximum concentration (*C*_max_), respectively. After repeated amcenestrant 400 mg dose QD administration, moderate total variability was observed on day 22 (coefficients of variation [CVs] for *C*_max_ and AUC_0-24h_ were 28.0% and 37.5%, respectively), apparent total clearance of the drug from plasma at steady state (CLss/F) was low (10.6 L/h), and geometric mean (CV) trough concentration (*C*_trough_) reached was 466 ng/mL (87.3%).

Amcenestrant excreted in urine over the 24-h interval after QD administration represented <0.1% of the administered dose, indicating no renal clearance of the parent drug.

Results for the 4β-hydroxycholesterol pre-/post-treatment geometric mean ratio were similar to those observed in Part A (Supplementary Table S6).

### Part B dose-expansion phase: secondary endpoints: antitumor activity

ORRs by baseline *ESR1* status (wild-type and mutated) were 15.4% and 5.3%, respectively, by ICR review; corresponding values for CBR were 34.6% and 21.1%, respectively (Table 4).

Median time to first response was 8.1 weeks by investigator/local radiologists review.

Tumor shrinkage occurred in 21/41 (51.2%) patients (Fig. 3a by ICR).

Duration of treatment up to 117 weeks were observed (Fig. 3b). The ORR by investigator/local radiologists review was 4/46 (8.7%) with a median time to first response of 8.1 weeks and with three patients still under treatment at the date of data cutoff. Clinical Benefit was achieved in 12/46 (26.1%) patients according to the investigator/local radiologists review.

### Part B dose-expansion phase: post hoc exploratory subgroup analyses of antitumor activity

Because most patients recruited to the study were heavily pretreated with up to eight lines of prior therapy in the advanced setting (76.9% ≥2 prior lines), 73.8% had received prior targeted therapies, and 47.7% prior fulvestrant, we conducted three post hoc exploratory analyses for the Part B primary endpoint of antitumor activity. Among patients who had received ≤3 prior lines of therapy in the metastatic setting (*n* = 27), the ORR was 18.5% and the CBR was 44.4%; among patients who had not received prior CDK4/6 inhibitors (*n* = 19), the ORR was 26.3% and CBR 52.6%; and among patients who had not received prior mTOR or CDK4/6 inhibitors or fulvestrant (*n* = 11), the ORR was 36.4% and CBR was 54.5% (Table 5).

**Table 5:** Post hoc subgroup analyses by <3 prior lines of therapy in the metastatic setting, by no prior CDK4/6 or mTOR inhibitors or fulvestrant, and by no prior CK4/6 inhibitors in the Part B response-evaluable population by independent central review.

	≤3 prior lines in the metastatic setting^d^	No prior CDK4/6i, mTORi, or fulvestrant^e^	No prior CDK4/6i^f^
Best overall response, *n* (%)			
Number	27	11	19
Complete response (CR)^a^	1 (3.7)	0	1 (5.3)
Partial response (PR)^a^	4 (14.8)	4 (36.4)	4 (21.1)
Stable disease (SD)	12 (44.4)	4 (36.4)	9 (47.4)
Progressive disease (PD)	10 (37.0)	3 (27.3)	5 (26.3)
Not evaluable	0	0	0
Objective response rate, *n* (%) [90% CI]^a,b^	5 (18.5) [7.6–35.1]	4 (36.4) [13.5–65.0]	5 (26.3) [11.0–47.6]
Clinical benefit rate, *n* (%) [90% CI]^c^	12 (44.4) [28.0–61.8]	6 (54.5) [27.1–80.0]	10 (52.6) [32.0–72.6]

*CDK4/6* cyclin-dependent kinase 4 and 6, *CI* confidence interval, *i* inhibitor, *mTOR* mammalian target of rapamycin.

^a^Confirmation of complete and partial responses are required (i.e., a second examination done ≥28 days apart, in order to confirm the antitumor response).

^b^90% CI estimated by the Clopper-Pearson interval.

^c^Clinical benefit rate: complete or partial responses or prolonged stable disease (i.e., for ≥24 weeks).

^d^Subset of Part B response-evaluable population with ≤3 prior lines in the metastatic setting, including ≤1 of either prior chemotherapy or CDK4/6 inhibitor, and no prior mTOR inhibitor.

^e^Subset of Part B response-evaluable population with no prior fulvestrant, CDK4/6 inhibitor, or mTOR inhibitor.

^f^Subset of Part B response-evaluable population with no prior CDK4/6 inhibitor.

### Post hoc exploratory biomarker analyses

In order to maximize the sample size and exposure to a pharmacodynamically active amcenestrant dose for post hoc exploratory biomarker analyses, we selected a pooled population of patients from Parts A and B who had received an amcenestrant dose ≥150 mg. We chose this dose cutoff because doses ≥150 mg showed high levels of ER occupancy on ^18^F-FES PET scans, whereas ER occupancy was very low for two of the three patients receiving amcenestrant 20 mg (Fig. 2b).

The biomarker population comprised 62 patients: 13 received amcenestrant QD ≥ 150 mg during Part A and 49 during Part B. Among these patients, median age was 63 (range 37–88) years, 59.7% had ECOG PS of 0, 93.5% had visceral metastases (Table 2), and the majority were heavily pretreated; the number of prior lines of therapy in the advanced setting ranged from 1 to 8 (48.4% had received ≥3 prior lines), all had received prior endocrine therapy, 72.6% had received prior targeted therapy, and 46.8% prior fulvestrant (Table 2).

**Fig. 4:**
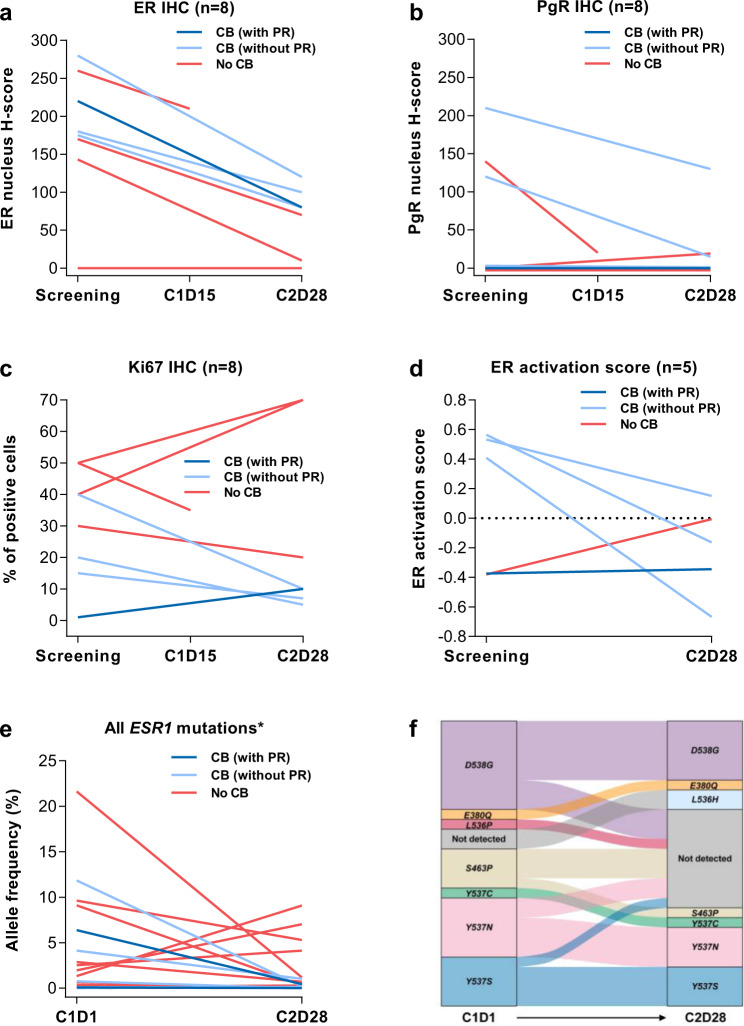
Post hoc exploratory biomarker analyses of on-target ER degradation/pathway inhibition during amcenestrant therapy. Changes from screening to cycle 2, day 28 in **a** ER nucleus H-score by IHC; **b** PgR nucleus H-score by IHC; **c** percent of positive cells showing Ki67 protein expression by IHC; and **d** ER activation scores by GSVA; and changes from cycle 1, day 1 to cycle 2, day 28 in **e** allele frequency of *ESR1* mutations in cfDNA by ddPCR; and **f** numbers of *ESR1* mutations in cfDNA by ddPCR. *Each line refers to an *ESR1* mutation, but several lines may refer to a single patient. C1D1, cycle 1, day 1; C1D15, cycle 1, day 15; C2D28, cycle 2, day 28; CB clinical benefit (complete response + partial response + stable disease ≥ 24 weeks), cfDNA circulating free DNA, ddPC, droplet digital polymerase chain reaction, ER estrogen receptor, *ESR1* estrogen receptor 1, GSVA gene set variation analysis, IHC immunohistochemistry, PgR progesterone receptor, PR partial response.

In ER degradation/pathway inhibition analyses, amcenestrant demonstrated robust on-target activity as shown by overall reductions in ER expression (ER degradation) (Fig. 4a; median relative change from screening was −58% [range −93 to −44%] among patients with non-zero values at screening and available data at cycle 2, day 28; *n* = 6), reduction in PgR expression (Fig. 4b; median relative change from screening was −88% [range −100 to −38%]; *n* = 3), reduction in Ki67 protein expression (Fig. 4c; median change from screening in percentage of Ki67 positive cells was −8% [range −30 to +30%]; *n* = 7), and reduction in ER activation score by gene set variation analysis (GSVA) (Fig. 4d; median change from screening was −0.4 [range −1.1 to +0.4]; *n* = 5). ER or PgR nucleus H-scores decreased at cycle 2, day 28 in all patients with non-zero screening values. Among patients with ER or PgR nucleus H-scores equal to zero at screening, one patient had an increase in PgR nucleus H-score at cycle 2, day 28. ER activation score decreased or remained stable only among patients who showed clinical benefit (median change from screening was −0.6 [range −1.1 to 0]; *n* = 4), whereas ER activation score increased for the patient with no clinical benefit (change from screening: +0.4).

Among response-evaluable patients in the biomarker population with *ESR1* mutations at cycle 1, day 1 (28/58; 48.3%) detected by multiplex droplet digital polymerase chain reaction (ddPCR) in circulating free DNA (cfDNA), the ORR was 1/28 (3.6%), and 9/28 (32.1%) patients treated with amcenestrant achieved clinical benefit, including in tumors with resilient *D538G* and *Y537S* mutations (Table 6).

**Table 6:** *ESR1* mutation status at baseline in patients from the biomarker population (Parts A and B excluding the pharmacodynamically inactive amcenestrant QD 20-mg dose) with available data for baseline *ESR1* mutational analysis in cfDNA detected by ddPCR (*n* = 58), and analyzed by those who subsequently achieved clinical benefit versus no benefit during amcenestrant therapy.

*ESR1* mutations	CB (*N* = 20)	No CB (*N* = 38)
Patients with *ESR1* wild-type	11	19
Patients with *ESR1* mutated	9	19
*D538G*	5/9 (55.6%)	11/19 (57.9%)
*Y537S*	4/9 (44.4%)	3/19 (15.8%)
*Y537N*	3/9 (33.3%)	4/19 (21.1%)
*L536H*	1/9 (11.1%)	0/19 (0.0%)
*S463P*	1/9 (11.1%)	5/19 (26.3%)
*Y537C*	1/9 (11.1%)	4/19 (21.1%)
*E380Q*	0/9 (0.0%)	5/19 (26.3%)
*L536P*	0/9 (0.0%)	1/19 (5.3%)
*L536R*	0/9 (0.0%)	1/19 (5.3%)

*CB* clinical benefit (complete response + partial response + stable disease ≥24 weeks), *cfDNA* circulating free DNA, *ddPCR* droplet digital polymerase chain reaction, *ESR1* estrogen receptor 1, *QD* once daily.

Among response-evaluable patients in the biomarker population with available data for ddPCR at cycle 1, day 1 and at cycle 2, day 28 (*n* = 31), 14/31 (45.2%) patients had at least one *ESR1* mutation at cycle 1, day 1 (total number of mutations = 27). Of these 14, 13/14 (92.9%) demonstrated decreases in allele frequency from cycle 1, day 1 to cycle 2, day 28 for at least one mutation during amcenestrant treatment for both patients with and without clinical benefit (Fig. 4e). Amcenestrant therapy decreased the number of most *ESR1* mutations (including known resilient alleles *D538G* and *Y537S*). At cycle 2, day 28, 10/27 (37.0%) of these mutations were no longer detectable (Fig. 4f).

## Discussion

In the current AMEERA-1 study, among postmenopausal women with metastatic ER+/HER2− breast cancer treated with amcenestrant ≥20 mg QD as monotherapy, we observed an absence of DLTs without reaching the maximum tolerated dose (MTD), a favorable safety profile, and a strong pharmacokinetic/pharmacodynamic relationship between plasma amcenestrant concentration and saturation of ER occupancy in multiple tumor sites by ^18^F-FES PET/CT imaging at doses ≥150 mg as well as evidence of antitumor activity. On this basis, amcenestrant 400 mg QD was chosen as the monotherapy RP2D for the dose-expansion phase.

Amcenestrant demonstrated overall median ER occupancy of 97% on ^18^F-FES PET/CT scans across all doses (*n* = 15) and in multiple tumor sites, including lymph nodes, lung, and bone. Median occupancy was 100% at the RP2D of amcenestrant 400 mg QD (*n* = 3; range 87–100%). In contrast, fulvestrant 500 mg has shown median ER occupancy levels of 85% (*n* = 16; range −60% to 99%), with lower levels of ER occupancy being associated with an increased risk of disease progression^25,30^. Although ^18^F-FES PET/CT imaging can assess ER occupancy to its ligand estrogen, it does not constitute formal proof that amcenestrant degrades ERs in tumors; however, our paired tumor biopsy analyses confirmed that amcenestrant achieved on-target activity (ER degradation/pathway inhibition) among women with metastatic ER+/HER2– breast cancer whose tumors carried multiple *ESR1* mutations (Fig. 4a–d). Of note, although all patients were ER+ at the diagnostic biopsy, which may have been conducted before enrollment or screening, some patients had ER nucleus H-scores of zero at the screening biopsy, which may have arisen because of ER degradation from prior exposure to fulvestrant or to tissue sampling bias.

Various *ESR1* mutations were detected at baseline, including *D538G*, *Y537S*, and *Y537N*. The proportion of patients with *ESR1* mutations at baseline in the current study (49.2%) was higher than that generally reported in advanced/metastatic breast cancer (36.4%)^10^, but was consistent with other published studies among patients with hormone-receptor-positive metastatic breast cancer receiving monotherapy with fulvestrant or oral SERDs where rates as high as 65% have been reported^35–38^. This could suggest that patients with difficult-to-treat *ESR1* mutations tend to be overrepresented in the clinical trial setting. On treatment at cycle 2 day 28, amcenestrant reduced the number of most of these *ESR1* mutations in cfDNA, and in 37% of cases, these mutations were no longer detectable, including known resilient *Y537S* and *D538G* mutations (Fig. 4f). Moreover, amcenestrant showed clinical benefit independent of *ESR1* mutation status, demonstrating activity in tumors with *ESR1* mutations known to be associated with endocrine resistance. Thus, mechanisms other than *ESR1* mutations appear to drive resistance to amcenestrant among these patients.

Amcenestrant is orally administered QD and has a favorable pharmacokinetic profile, whereas fulvestrant, the only approved SERD, has low oral bioavailability and must be administered as two large-volume intramuscular injections with up to 1 month to reach a steady state^28^. Amcenestrant showed limited accumulation; and exposure increased proportionally with doses up to 600 mg after repeated oral administration. At the 400 mg dose, exposure showed moderate total variability (<40%) associated with a low systemic clearance (10.6 L/h), and no elimination in the urine. The increase in exposures when administered with food was considered a moderate effect when compared with the larger overall variability of pharmacokinetic exposures, allowing amcenestrant to be administered regardless of food status. The mean *C*_trough_ concentration obtained at the 400 mg dose level (geometric mean 466 ng/mL, Supplementary Table S8) was well above the minimum concentration associated with near 90% occupancy or more of ERs (Fig. 2b). This *C*_trough_ was about 40 times higher (in molar concentration) than that for fulvestrant (12.5 ng/mL) at steady state after 500 mg intramuscular administration (day 1, day 14 of cycle 1, 500 mg monthly from cycle 2)^39^.

In dose expansion, the primary endpoint evaluating antitumor activity showed an ORR by ICR of 5/46 (10.9%), comprising 1 CR and 4 PRs, and a CBR of 13/46 (28.3%). It is important to note that most patients were heavily pretreated with up to eight lines of therapies in the advanced setting, including up to six lines of hormonal therapies, and prior treatment with chemotherapy, fulvestrant, and cyclin-dependent kinase 4 and 6 (CDK4/6), phosphoinositide 3-kinase (PI3K), and mammalian target of rapamycin (mTOR) inhibitors.

Although cross-trial comparisons should be conducted with caution and the current trial sample size was small, we indirectly compared ORR and CBR with methodologically similar studies investigating the recommended 500 mg dose of fulvestrant monotherapy in the second or third-line setting among postmenopausal women (or where pre- or perimenopausal women had received a gonadotropin-releasing hormone analog prior to randomization that continued throughout the study) with advanced/metastatic ER+/HER2− breast cancer and whose tumor responses were confirmed as per RECIST v1.1 in populations with measurable disease. Four studies met these criteria. The PALOMA-3^40^ and FAKTION^41^ studies recruited women who had received up to three prior lines of endocrine therapy and the SOLAR-1^42^ and SANDPIPER^43^ studies with up to two prior lines. The PALOMA-3 study excluded women who had received prior fulvestrant and CDK4/6, PI3K, and/or mTOR inhibitors. The FAKTION, SOLAR-1, and SANDPIPER studies excluded women who had received prior fulvestrant and PI3K inhibitors, and the SOLAR-1 and SANDPIPER studies also excluded women who had received prior mTOR inhibitors. All four comparator studies investigated less heavily pretreated patients than those in AMEERA-1. In these studies, the fulvestrant monotherapy ORR ranged from 10.9 to 16.2% and CBR ranged from 36.0 to 44.1%^40–43^.

Our post hoc exploratory subgroup analyses assessed the impact of prior therapies on antitumor activity with amcenestrant. Among women who had received ≤3 prior advanced lines in the metastatic setting (*n* = 27), the ORR was 18.5% and CBR was 44.4%, which appeared numerically comparable to historical results with fulvestrant monotherapy^40–43^. Among women who had not received prior CDK4/6 inhibitors (*n* = 19), the ORR was 26.3% and CBR 52.6%. Among women who had not received prior CDK4/6 or mTOR inhibitors or fulvestrant (*n* = 11), the ORR was 36.4% and CBR was 54.5%. Given that endocrine therapy plus a CDK4/6 inhibitor is now standard of care for the first-line management of patients with ER+/HER2− advanced/metastatic breast cancer^44^, which was not established practice at the time AMEERA-1 was designed, we recognize that patients who had not received a prior CDK4/6 inhibitor (35.4% in the current study) had a greater antitumor response to amcenestrant than patients who had received a prior CDK4/6 inhibitor. However, because AMEERA-1 was a first-in-human Phase 1 study, it was important to evaluate the safety and antitumor activity of amcenestrant in a broad population of patients with advanced/metastatic breast cancer regardless of prior therapy use.

Amcenestrant demonstrated a favorable safety profile and treatment was well-tolerated. No TEAEs led to treatment discontinuation and most were Grade 2 or less. No serious AEs were considered treatment-related by investigators and no treatment-related TEAEs of Grade ≥3 occurred. Among listed serious AEs (Supplementary Table S7), no clinically significant cardiac or eye safety findings were observed, whereas these AEs have been reported with other orally administered SERDs in development^23,24^.

Study strengths include the combined evaluation of safety, antitumor activity, and pharmacokinetics/pharmacodynamics demonstrating robust target engagement and ER degradation/pathway inhibition in ER+/HER2− advanced breast cancer. Study limitations of this Phase 1/2 trial include the small sample size because the prespecified interim analysis results after at least four cycles of amcenestrant therapy led to stopping accrual to Part B. With continued follow-up and monitoring, five patients had responded at the final analysis cutoff date of 30 March 2021. Had these responses been observed earlier than the cutoff date of 30 May 2020, Part B accrual would have continued. However, the knowledge that amcenestrant could be associated with ongoing responses after prolonged therapy was lacking at the time of the design of the study. Given that prolonged responses with amcenestrant were observed, including encouraging clinical benefit rates, and with a very promising safety profile, we considered it unnecessary to reopen the study because sufficient information on the compound had been gained to justify continuing with the amcenestrant clinical development program in Phase 2 and Phase 3 trials. Additional limitations included, a lack of racial diversity, not all patients having paired tumor biopsies, and the absence of a control arm, requiring validation of these results in larger comparative studies.

Amcenestrant development plan studies in specific populations include: the ongoing AMEERA-1 investigating amcenestrant in combination with palbociclib (Parts C and D), alpelisib (Parts F and G), everolimus (Parts H and I), or abemaciclib (Parts J and K); AMEERA-3 (NCT04059484), a randomized Phase 2 trial investigating amcenestrant monotherapy versus physician’s choice of endocrine therapy among patients with ER + /HER2 − locally advanced or metastatic breast cancer with prior exposure to hormonal therapies; AMEERA-4 (NCT04191382), a window of opportunity study investigating amcenestrant at two dose levels versus letrozole on change in Ki67 (percentage of positive tumor cells) among newly diagnosed preoperative postmenopausal women with ER+/HER2− breast cancer; and AMEERA-5 (NCT04478266), a randomized, multicenter, double-blind, Phase 3 study, investigating amcenestrant plus palbociclib versus letrozole plus palbociclib among patients with ER+/HER2− breast cancer who have not received prior systemic anti-cancer treatment for advanced disease.

In conclusion, amcenestrant, an oral SERD, at RP2D of 400 mg QD for monotherapy showed no DLTs and a favorable safety profile with no clinically significant cardiac or eye safety findings, and demonstrated preliminary antitumor activity irrespective of baseline *ESR1* mutation status among postmenopausal women with metastatic ER+/HER2− breast cancer, most of whom were heavily pretreated with targeted therapies and/or fulvestrant. Amcenestrant exhibited pharmacodynamic evidence of ER degradation/pathway inhibition on biomarker analysis with functional imaging and paired tumor biopsies, and decreased *ESR1* mutations, including known resilient alleles *D538G* and *Y537S*.

## Methods

### Study design

AMEERA-1 (NCT03284957) is a multinational, open-label, nonrandomized, dose-escalation and dose-expansion, safety, antitumor activity, pharmacokinetics, and pharmacodynamic study consisting of five arms, each with two parts as follows: Arm 1, amcenestrant administered orally as monotherapy in 28-day cycles in dose escalation (Part A) and dose expansion (Part B) cohorts; Arm 2, amcenestrant combined with palbociclib in dose escalation (Part C) and dose expansion (Part D) cohorts; Arm 3, amcenestrant combined with alpelisib in safety run-in (Part F) and dose expansion (Part G) cohorts; Arm 4, amcenestrant plus everolimus in dose-escalation (Part H) and dose expansion (Part I) cohorts; and Arm 5, amcenestrant plus abemaciclib in dose-escalation (Part J) and dose expansion (Part K) cohorts. Here, we describe the final analysis results with amcenestrant monotherapy from Arm 1 (Parts A and B). Clinical data were collected in electronic case report forms using Medidata Rave v2020.3.2.

### Patients

The first patient was enrolled on 6 November 2017 and the last patient was enrolled on 26 March 2020. Key inclusion criteria were as follows: postmenopausal women; histological or cytological proven diagnosis of ER+/HER2− breast adenocarcinoma; measurable disease by RECIST v1.1; ≥6 months prior endocrine therapy for advanced breast cancer; ≤3 prior chemotherapeutic regimens for dose escalation eligibility and ≤1 prior chemotherapeutic regimen for dose expansion eligibility; ECOG PS 0 or 1. Key exclusion criteria were as follows: known brain metastases, leptomeningeal carcinomatosis, and/or spinal cord compression; prior treatment with another SERD, except fulvestrant following a ≥6-week washout period. Full criteria are presented in the online Supplementary Information (Supplementary Methods: Full inclusion exclusion criteria). The protocol was approved by the institutional review board or independent ethics committee at each site (see Supplementary Table S9 for the names of the boards/committees who provided approval) and complied with the International Ethical Guidelines for Biomedical Research Involving Human Subjects, Good Clinical Practice guidelines, the Declaration of Helsinki, and local laws. All patients provided written informed consent and no compensation was offered.

### Treatment

During dose escalation, treatment was initiated on day 1 in the fasting state in cohorts starting at a dose of 20 mg orally QD, escalating in subsequent cohorts to planned doses of 50–600 mg. For all cohorts, treatment was omitted on day 2, was administered on day 3 after a moderate fat breakfast, and was administered from day 4 in the fasting state. Dose escalation proceeded based on the occurrence of DLT together with target saturation by ^18^F-FES PET/CT scans^45^ and pharmacokinetic parameters during the first 28-day cycle, and according to a 3 + 3 design. For a detailed description of methods for dose escalation and selection of the RP2D, see Supplementary Methods: Dose escalation amcenestrant monotherapy. A twice-daily (BID) dosing regimen of 6 patients receiving amcenestrant 300 mg BID was also planned. During dose expansion, patients were treated at the RP2D. Patients were treated until disease progression, unacceptable toxicity, withdrawal of patient consent, or other reasons determined by the investigator (poor compliance with the protocol or intercurrent illness).

Treatment exposure was assessed using relative dose intensity (%), defined as (actual dose intensity ÷ planned dose intensity) × 100 where actual dose intensity was defined as the total cumulative dose in mg ÷ ((duration of treatment in weeks × 7) − 1) and planned dose intensity in mg/day was defined as the planned dose at cycle 1 day 1.

### Primary endpoints

In Part A, the dose-escalation phase, the primary endpoints were the incidence of treatment-related DLTs during cycle 1 (days 1–28), defined as the occurrence of any TEAEs related to the study therapy from a predefined list of hematological, non-hematological, and hepatic AEs as per the National Cancer Institute Common Terminology Criteria for Adverse Events (NCI-CTCAE) v4.03 (see Supplementary Methods: Definition of DLTs for a list of DLT-defining TEAEs); and the determination of the MTD, defined as the highest dose level at which ≤1/6 evaluable patients experienced a DLT, and the RP2D.

In the absence of DLTs, the RP2D was defined considering pharmacokinetics after repeated administration, level of inhibition of target occupancy measured by ^18^F-FES PET/CT imaging, and pharmacokinetics/pharmacodynamics on ER occupancy. The RP2D should be at least two dose levels above the dose level showing >90% decrease in maximum standardized uptake value (average of all index lesions’ maximum ^18^F-FES uptake standardized by body weight and corrected for background ^18^F-FES uptake: SUV_max(cor)_) on ^18^F-FES PET/CT imaging (considered to represent inhibition of estrogen ligand binding or near-complete ER degradation), unless there were DLTs at this dose, in which case the RP2D could be any dose where >90% decrease in standardized uptake was reached.

In Part B, the dose-expansion phase, the primary endpoint was the confirmed ORR by ICR.

Because most patients recruited to the study were heavily pretreated with up to 8 lines of prior therapy in the advanced setting (76.9% ≥2 prior lines), 73.8% had received targeted therapies, and 47.7% prior fulvestrant, we conducted two exploratory subgroup analyses for the Part B primary endpoint among patients who had received ≤3 prior lines of therapy in the metastatic setting, and among patients who had not received prior CDK4/6 or mTOR inhibitors or fulvestrant. These subgroup analyses based upon prior treatments were performed to match baseline patient characteristics to historical studies with fulvestrant for indirect antitumor activity comparison. In addition, we conducted a third exploratory subgroup analysis among patients who had not received a prior CDK4/6 inhibitor.

### Secondary endpoints

Overall safety in Parts A and B was assessed by tabulating TEAEs, coded using the Medical Dictionary for Regulatory Activities (MedDRA) version 22.1, and graded according to NCI-CTCAE v4.03 criteria. TEAEs were classified as related or unrelated to amcenestrant therapy based upon the local investigating physician’s clinical judgment.

To evaluate pharmacokinetics in Part A, venous blood samples were collected pre-dose and at regular intervals on cycle 1, day 1 (up to 48 h), on day 3 (up to 24 h), and on day 22 (up to 24 h). Amcenestrant concentrations were determined in plasma using a validated liquid chromatography-tandem mass spectrometry method. The validated range was 5 ng/mL to 5000 ng/mL. Plasma pharmacokinetic parameters, including *t*_*max*_, *C*_max_, and AUC_0-24h_, were determined after a single dose on cycle 1 day 1 in the fasting state and on day 3 in the fed state (moderate fat breakfast). After multiple-dose administration, *t*_max_, *C*_max_, AUC_0-24h_, *C*_trough_, and CLss/F were determined on day 22 in the fasting state.

In Part B, venous blood samples were collected pre-dose and at regular intervals on cycle 1, day 1 (up to 24 h), and on day 22 (up to 24 h). Plasma pharmacokinetic parameters (*t*_max_, *C*_max_, AUC_0-24h_ on day 1 and day 22, and in addition, *C*_trough_ and CL_ss_/F on day 22) were determined regardless of food status in a subset of patients participating with repeated pharmacokinetic sampling. On day 22, 24 h urine fraction was collected to determine the amount of amcenestrant eliminated by renal clearance.

The potential induction/inhibition effect of amcenestrant on CYP3A using 4β-hydroxycholesterol was assessed for both Parts A and B.

To evaluate pharmacodynamics in Part A, ^18^F-FES PET/CT imaging was performed at baseline and between Days 11 and 15 of the first cycle (16–24 h after the previous amcenestrant dose) to evaluate the extent of residual ER availability at a steady state of ER occupancy (after ≥8 continuous days of treatment). ER occupancy was calculated as the percentage reduction in ^18^F-FES SUV_max(cor)_ between the on-treatment and screening scans. In addition, a blood sample was collected just before ^18^F-FES radioisotope administration on the day of ^18^F-FES PET/CT scan (day 11–15) to assess the correlation between the plasma concentration of amcenestrant and scan results. For detailed ^18^F-FES PET/CT imaging and image analysis methods see Supplementary Methods: ^18^F-FES PET/CT imaging and image analysis.

In Part A, preliminary antitumor activity was assessed using the ORR, defined as a complete response or partial response, confirmed on a second examination at least 4 weeks apart, as per RECIST v 1.1, as well as the CBR (CBR = confirmed CR, PR, or SD ≥ 24 weeks) by investigator/local radiologist review.

In Part B, the ORR and CBR by baseline *ESR1* gene mutational status (wild-type and mutated), overall ORR and CBR by investigator/local radiologists review as well as time to first tumor response (CR or PR) were assessed.

### Post hoc analyses

Post hoc exploratory biomarker analyses were conducted. In order to maximize sample size and exposure to a pharmacodynamically active amcenestrant dose for exploratory biomarker analyses, we selected a pooled population of patients from Parts A and B who had received an amcenestrant dose ≥150 mg. We chose this dose cutoff because doses ≥150 mg showed high levels of ER occupancy on ^18^F-FES PET scans, whereas ER occupancy was very low for two of the three patients receiving amcenestrant 20 mg (Fig. 2b).

To analyze ER degradation/pathway inhibition, tumor biopsies were collected at screening and at the end of cycle 2 (cycle 2, day 28) in patients from Part B who provided consent for their collection. For each biopsy, formalin-fixed paraffin-embedded (FFPE) tissue sections were collected of 5 µm each for immunohistochemistry (IHC) analysis, and of 10 µm each for RNA extraction and subsequent RNA-seq analysis. IHC ER, PgR, and Ki67 protein expression slides were assessed by two expert pathologists (V.P. and A.-L.B.). IHC staining was performed on the Ventana Discovery XT IHC platform using anti-ER clone SP1 (CONFIRM Anti-Estrogen Receptor, Roche, ref. 790–4325), anti-PgR clone 1E2 (CONFIRM Anti-progesterone receptor, Roche, ref. 790–4296), and anti-Ki67 clone 30-9 (CONFIRM Anti-Ki-67, Roche, Ref. 790–4286). Stained slides were evaluated by standard light microscopy using a manual scoring system. Staining was evaluated in tumor cells only. For ER and PgR expression, H-scores were calculated^46^. For Ki67 protein expression, the percentage of positive tumor cells was calculated.

Total RNA was extracted from FFPE tissue (slides of 10 µm). A targeted-enrichment RNA-seq approach was applied utilizing KAPA RNA HyperPrep and whole exome SeqCap kits (Roche) followed by the generation of sequencing reads with NextSeq 500 (Illumina). RNA-seq FASTQ files were processed with STAR aligner and Cufflinks to generate gene-level fragments per kilobase of transcript per million mapped reads (FPKM)^34,47,48^. Those FPKM values were converted to the gene-level estimation of expression in transcripts per million (TPM). The TPM data were then quantile-normalized and log2-transformed. GSVA was employed to calculate the ER activation score by using the ER activity signature (87 genes)^31^.

To evaluate *ESR1* mutations, plasma samples were collected at cycle 1, day 1 and at the end of cycle 2, day 28 to assess *ESR1* mutations. Wild-type and mutant *ESR1* status in cfDNA was determined by ddPCR detecting 12 *ESR1* pathogenic single-nucleotide variants in the ligand-binding domain of *ESR1* using the OncoBEAM™ platform by Sysmex Inostics (Baltimore, MD, USA)^49^.

### Statistical considerations

Sample size considerations. A minimax Simon 2-stage design with a 5% one-sided Type 1 error rate was used to test the null hypothesis of a 10% response rate. Under the alternative assumption of 20% response rate, a total of 78 patients were needed to guarantee at least 80% power, accounting for the binding futility interim analysis. In the first stage, 45 patients were to be accrued in Part B and were evaluated for tumor responses after they had had two tumor assessments (i.e., after at least four cycles of amcenestrant therapy) or early progression or an end-of-treatment tumor assessment, whichever occurred first. If ≤4 responders by ICR in these 45 patients were observed, accrual to Part B would be stopped. The null hypothesis would be rejected if ≥13 responders were observed in the 78 patients.

Analysis populations and associated statistical methods. The DLT-evaluable population during Part A was planned to include all patients who had received a first complete cycle, who had received at least 75% of the intended dosing (unless the patient discontinued treatment before cycle 1 completion because of a DLT), and had evaluable ^18^F-FES PET/CT scans at baseline and between day 11 and day 15 of the first cycle. Results were analyzed using descriptive statistics.

The safety population included all patients exposed to at least one dose of the study treatment. Results were analyzed using descriptive statistics.

The response-evaluable population was defined as treated patients with measurable disease at study entry who had an evaluable baseline and at least one post-baseline evaluable tumor assessment. Patients with an early progression as per RECIST v1.1 or who died from disease progression were evaluable for response. Results were analyzed using mostly descriptive statistics; for ORR and CBR, 90% CIs were calculated using the exact method of Clopper-Pearson.

The pharmacokinetic population evaluable for non-compartmental pharmacokinetic analysis (NCA) was defined as all patients from the all-treated population without any major deviations related to study treatment administration (e.g., early vomiting just after drug administration, food status), and who had adequate blood samples enabling determination of at least one pharmacokinetic parameter. NCA was performed using Phoenix^®^ software.

The biomarker-evaluable population was defined as all patients with available biomarker data from the response-evaluable pooled population of patients from Parts A and B who had received an amcenestrant dose ≥150 mg excluding the pharmacodynamically inactive 20-mg dose. Only paired samples (screening or cycle 1, Day1 and available cycle 2, day 28 samples) were considered for assessment of the evolution of biomarkers over time. Results were analyzed using descriptive statistics.

### Reporting summary

Further information on research design is available in the Nature Research Reporting Summary linked to this article.

## Supplementary information

Supplementary Information

Reporting Summary

## Data Availability

Researchers, such as any researcher holding a faculty appointment or research position at an institution of higher education, a research organization, or a nonprofit organization, may request access to patient-level data and related study documents including the clinical study report, study protocol with any amendments, blank case report form, statistical analysis plan, and dataset specifications for legitimate research purposes, as evaluated by an independent scientific review board. Patient-level data will be anonymized and study documents will be redacted to protect the privacy of our trial participants. Access to data may be restricted in cases where data collected are subject to contractual obligations (for example, data obtained by Sanofi from independent institutions where the data sharing contract prohibits onward sharing to third parties) or require patient consent for sharing (for example, if patients do not consent for their data to be shared with third parties or to be deposited in repositories). Data use agreements will be used for approved data requests. Access to data is expected to take on average 2–5 months, and will depend on various factors. These factors include the number of data contributors, the number of studies, the availability of the requestor to respond to comments, the ability to align with the data use agreement, and if the data from the trial have already been anonymized. Further details on Sanofi’s data-sharing criteria, eligible studies, and process for requesting access can be found at: https://www.vivli.org/. Patients in this study did not give consent for their genomic data to be shared or deposited in repositories. Thus, in accordance with Sanofi’s commitment to honoring patient preferences and protecting patient privacy, patient-level genomic data, including RNA-seq data, are not available. Although Parts A and B of this study are complete, which comprise the data presented in this manuscript, the other parts of the study (Parts C through K) are ongoing. As such, at the time of publication, the study is not eligible for posting on the Vivli platform until all parts of the study have been completed. However, requests for data of unlisted studies can be placed via the following link: https://vivli.org/members/enquiries-about-studies-not-listed-on-the-vivli-platform/.
