# Supplementary material for: AMEERA-1 phase 1/2 study of amcenestrant, SAR439859, in postmenopausal women with ER-positive/HER2-negative advanced breast cancer
Source: Nat Commun. 2022 Jul 15;13:4116. doi: 10.1038/s41467-022-31668-8 (PMC9284491; doi:10.1038/s41467-022-31668-8)
Supplement: Supplementary file 1 — Supplementary Information [file 41467_2022_31668_MOESM1_ESM.pdf]

Supplementary Information

AMEERA-1 Phase 1/2 study of amcenestrant, SAR439859, in postmenopausal women with ER-positive/HER2-negative advanced breast cancer

Aditya Bardia[1,12], Sarat Chandarlapaty[2,12], Hannah M. Linden[3,12], Gary A. Ulaner[4,5], Alice Gosselin[6], Sylvaine Cartot-Cotton[6], Patrick Cohen[6], Séverine Doroumian[7], Gautier Paux[8], Marina Celanovic[8], Vasiliki Pelekanou[8,11], Jeffrey E. Ming[9], Nils Ternès[6], Monsif Bouaboula[8], Joon Sang Lee[8], Anne-Laure Bauchet[6], Mario Campone[10,12] ✉.

[1]Massachusetts General Hospital Cancer Center, Harvard Medical School, Boston, MA, USA. [2]Memorial Sloan Kettering Cancer Center, New York, NY, USA. [3]University of Washington Medical Center, Seattle Cancer Care Alliance, Seattle, WA, USA. [4]Hoag Family Cancer Institute, Newport Beach, CA, USA. [5]University of Southern California, Los Angeles, CA, USA. [6]Sanofi, Paris, France; [7]Sanofi, Montpellier, France; [8]Sanofi, Cambridge, MA, USA; [9]Sanofi, Bridgewater, NJ, USA; [10]Institut de Cancérologie de l'Ouest, René Gauducheau, Saint-Herblain, France. [11]Present address: Bayer US-Pharmaceuticals, Cambridge, MA, USA. [12]These authors contributed equally: Aditya Bardia, Sarat Chandarlapaty, Hannah M. Linden, Mario Campone. ✉email: Mario.Campone@ico.unicancer.fr

Supplementary Notes: BID dosing regimen

Patient disposition

Of 6 recruited participants who were treated with amcenestrant 300 mg BID, 2 were evaluable for DLTs and 4 were evaluable for tumor response. All 6 patients discontinued study treatment, 5 due to progressive disease and 1 due to an adverse event.

Protocol violations

Of 6 recruited participants, 4 showed critical or major protocol violations, including two patients who could not have FES PET scans performed due to the coronavirus pandemic.

Patient demographic and baseline characteristics

Mean age was 59.7 (SD ±13.7) years. The oldest participant was 86 years old and the youngest participant was 47 years old. ECOG status was 1 in 50% of the participants and ECOG 0 in the remaining participants. Mean baseline weight was 73.47 (SD ±15.18) kg

The median time from first diagnosis to first study treatment administration was 10.41 years (range 5.7 to 14.3). One (16.7%) participant had a Stage II disease at diagnosis, 2 (33.3%) had Stage III, 1(16.7%) had Stage IV, and the remaining 2 (33.3%) participants were unknown. The histopathology type was moderately differentiated for 4 participants, and unknown for 2 participants

All 6 treated participants had received at least 1 prior treatment in advanced setting with 1 (16.7%) participants pretreated with 3 prior lines and 5 (83.3%) participants pretreated with >3 prior lines. All participants received prior chemotherapy, hormonotherapy, and targeted therapy in advanced settings.

Treatment exposure

The median duration of treatment for BID dosing was 8.0 weeks (ranging from 2–32 weeks). The average relative dose intensity was 82.3% (± 16.3).

Safety

No dose-limiting toxicities occurred. All 6 participants had at least 1 TEAE regardless of the relationship to amcenestrant. The most frequently reported TEAEs in 2 or more of the 6 participants were diarrhea (n=3;

50%), and vomiting, nausea, and rash (n=2 each; 33.3%). No deaths were reported. One participant had a treatment emergent SAE (Grade ≥3) of hypophosphatemia that led to definitive treatment discontinuation.

Antitumor activity

Four participants were evaluable as per RECISTv.1.1. Best overall response was stable disease in 1 (25%) participant and progressive disease in 3 (75%) participants.

Supplementary Methods

Dose escalation amcenestrant monotherapy

Dose escalation was initiated with a once daily (QD) schedule with a starting dose of 20 mg/day. Dose escalation was expected to proceed according to the schedule shown in Table 1 of the main manuscript. Intra-patient dose escalation was not permitted.

Part A of this study was designed using the 3 + 3 concept; 3 to 6 patients were treated at each dose level depending on dose-limiting toxicities (DLTs) observed in the first 3 patients. If one of the first 3 evaluable patients experienced DLTs during Cycle 1, this cohort was expanded with a total of up to 6 patients. If less than 1 out of 3 patients or less than 2 out of 6 patients experienced DLTs at a given dose level, the dose escalation proceeded to the next dose level.

In addition, ^{18}F-FES PET/CT scan results were available for all DLT-evaluable patients and depending on results at DL1 and DL2, the intermediate dose levels (DL1bis and DL2bis) were explored.

The following rules regarding ^{18}F-FES PET/CT scan findings and dose levels to be tested were applied:

• If all patients treated at DL1 had 100% of inhibition of the target as shown on ^{18}F-FES PET/CT scan, DL(-1) was explored.

• If all patients treated at DL1 had >70% inhibition of the target as shown on ^{18}F-FES PET/CT scan results, DL1bis was explored.

• If at least one patient at DL1 had between 30% and 70% inhibition of the target as shown on ^{18}F-FES PET/CT scan results, dose escalation continued as planned and DL2 was explored.

• If at least one patient at DL1 had ≤30% inhibition of the target as shown on ^{18}F-FES PET/CT scan results, DL2bis was explored.

• If all patients treated at DL2 had >85% inhibition of the target as shown on ^{18}F-FES PET/CT scan results, DL2bis was explored.

• If at least one patient at DL2 had ≤85% inhibition of the target as shown on ^{18}F-FES PET/CT scan results, dose escalation continued as planned and DL3 was explored.

From the 2 dose levels DL1bis and DL2bis, the next dose levels (DL2 and DL3 etc. respectively) were not skipped.

At subsequent dose levels (≥ DL3), other intermediate or higher dose levels were tested based on safety, ^{18}F-FES PET/CT scan results (if all patients had >90% inhibition of the target) and pharmacokinetic parameters upon recommendation from the study committee.

The second and third patients of a given cohort were only enrolled when the first patient had received at least 1 week of amcenestrant without DLT. The enrolment at the next dose level did not proceed before at least 3 patients treated at the current dose level had been followed for at least 1 cycle duration (i.e., 28 days) and were evaluable for DLT assessment.

Patients who discontinued study treatment prematurely before the end of the DLT observation period for any reason other than DLT were replaced.

As a rule, the dose escalation stopped when the maximum administered dose (MAD) was reached; MAD was defined as the dose at which ≥33% (2 patients out of up to 6) of evaluable patients had experienced a DLT during Cycle 1.

The maximum tolerated dose (MTD) was defined as the highest dose level at which no more than 1 patient of a maximum of 6 patients experienced a DLT. Usually, the MTD is one dose level below the MAD or the highest dose tested if the MAD is not reached.

Although the dose escalation process is guided by the safety evaluation during Cycle 1 of treatment, cumulative or irreversible toxicities observed after subsequent administrations were also considered for the dose escalation and dose selection decisions (i.e., expansion of a given dose level, intermediate dose levels), as well as any other relevant information, upon recommendation from the study committee.

The recommended Phase 2 dose (RP2D) for the expansion cohort was primarily based on safety data, but also on pharmacodynamic (target saturation), pharmacokinetic and pharmacokinetic/pharmacodynamic data. If the MTD could not be determined in the absence of DLT at the MAD, pharmacokinetics after repeated administration, level of inhibition of target occupancy measured by ^{18}F-FES PET/CT imaging and pharmacokinetics/pharmacodynamics on ER occupancy as well as any other relevant information was taken into account to select the RP2D and for the decision to expand the study to Part B. The RP2D was at least 2 dose levels above the dose level showing >90% inhibition of the target on ^{18}F-FES PET/CT scan at this dose level, unless there were DLTs at this dose, in which case the RP2D was any dose level at which >90% inhibition was reached.

Full inclusion exclusion criteria

INCLUSION CRITERIA

Parts A and B

1) Patients must be postmenopausal women as defined by one of the following:
    a) Women >60 years.
    b) Women ≤60 years:
        i) With spontaneous cessation of menses >12 months prior to registration in the absence of chemotherapy, tamoxifen and toremifene.
        ii) Or with cessation of menses of duration ≤12 months or secondary to hysterectomy AND have follicle stimulating hormone (FSH) level in the postmenopausal range according to institutional standards (or >34.4 IU/L if institutional range is not available) prior to registration.
        iii) Or who have received hormonal replacement therapy but have discontinued this treatment AND have FSH level in the postmenopausal range according to institutional standards (or >34.4 IU/L if institutional range is not available) prior to registration.
        iv) Or with status post bilateral surgical oophorectomy.
        v) Or are premenopausal women on a gonadotropin-releasing hormone analog for at least 6 months (to be continued during study treatment) and have a negative pregnancy test prior to initiation of study treatment and at monthly intervals during treatment.
2) Patients with histological or cytological proven diagnosis of the breast adenocarcinoma with evidence of either locally advanced not amenable to radiation therapy or surgery in a curative intent, inoperable and/or metastatic disease.
3) Either the primary tumor or any metastatic site must be positive for estrogen receptor (ER) (>1% tumor cell staining by immunohistochemistry [IHC]).
4) Either the primary tumor or any metastatic site must be human epidermal growth factor receptor 2 (HER2) non-overexpressing by IHC (0, 1+), or in situ hybridization-negative based on single-probe average HER2 copy number <4.0 signals/cell or dual-probe HER2/centromeric probe for chromosome

17 (CEP17) ratio <2 with an average HER2 copy number <4.0 signals/cell as per American Society of Clinical Oncology guidelines (J Clin Oncol. 2013;31(31):3997-4013).

5) Prior chemotherapy for advanced/metastatic disease is allowed:
   a) Patients must have received no more than 3 prior chemotherapeutic regimens in Part A (dose escalation, monotherapy).
   b) Patients must have received no more than 1 prior chemotherapeutic regimen in Parts B, C and D (dose expansion monotherapy, dose escalation and expansion, combination with palbociclib). (NOTE: Antibody drug conjugates are considered as chemotherapy in this study).
6) Patients must have received at least 6 months of prior endocrine therapy for advanced breast cancer.
7) Age ≥18 years old.
8) Measurable lesion by Response Evaluation Criteria in Solid Tumors v1.1.
9) The patient is capable of understanding the informed consent and complying with the protocol and has signed the informed consent form before participation (specific screening procedures or evaluations).

Part A only

10) Patient entering Part A must agree to undergo:
    a) Two ^{18}F-fluoroestradiol positron emission tomography/computerized tomography (^{18}F-FES PET/CT) imaging scans, one at baseline and one between Day 11 and Day 15 of study treatment intake and
    b) Two ^{18}F-fluorodeoxyglucose (^{18}F-FDG) PET/CT imaging scans, one at baseline and one between Day 11 and Day 15 of study treatment intake before ^{18}F-FES PET (patients whose baseline ^{18}F-FDG PET/CT results are negative for tumors will not be eligible), and
    c) Paired biopsies (before treatment and during treatment): For baseline samples, formalin-fixed and paraffin-embedded (FFPE) archived biopsy samples (within past 3 months prior to the initiation of study treatment) can be used, but preferably fresh biopsies from primary tumor or recurrence or metastasis will be collected. It is recommended that the end of Cycle 2 biopsy (on-treatment biopsy) be collected at the same location as the baseline biopsy whenever the tumor is accessible and during treatment.

Part B only

11) For patients who consent to paired biopsies (before treatment and during treatment): for baseline samples, a FFPE archived biopsy sample can be used (within past 3 months prior to the initiation of study treatment) but preferably fresh biopsies from primary tumor or recurrence or metastasis will be collected. It is recommended that the end of Cycle 2 biopsy (on-treatment biopsy) be collected at the same location as the baseline biopsy whenever the tumor is accessible during treatment.

EXCLUSION CRITERIA

Patients who have met all the above inclusion criteria will be screened for the following exclusion criteria:

Exclusion criteria related to study methodology

1) Eastern Cooperative Oncology Group (ECOG) performance status ≥2.
2) Significant concomitant illness, including psychiatric condition that, in the opinion of the Investigator or Sponsor, would adversely affect the patient's participation in the study.
3) Medical history or ongoing gastrointestinal disorders potentially affecting the absorption of amcenestrant and/or palbociclib. Patients unable to swallow normally and to take capsules. Predictable poor compliance to oral treatment.
4) Any malignancy related to human immunodeficiency virus; or unresolved viral hepatitis.
5) Patients with a life expectancy less than 3 months.
6) Patients not suitable for participation, whatever the reason, as judged by the Investigator, including medical or clinical conditions, or patients potentially at risk of noncompliance to the study procedures (i.e., unwillingness and inability to comply with scheduled visits, drug administration plan, laboratory tests, other study procedures, and study restrictions).
7) Major surgery within 4 weeks prior to first study treatment administration.
8) Patient with any other cancer. Adequately treated basal cell or squamous cell skin cancer or in situ cervical cancer or any other cancer from which the patient has been disease free for >3 years are allowed.

9) Patient is the Investigator or any subinvestigator, research assistant, pharmacist, study coordinator, or other staff or relative thereof directly involved in the conduct of the protocol.
10) In Part A only: Patient with liver metastases only.

Exclusion criteria related to the disease

11) Patients with known brain metastases, leptomeningeal carcinomatosis and/or spinal cord compression. Patients with brain metastases that have been previously totally resected or irradiated are eligible provided no progression or relapse is observed within 4 weeks of the treatment.
12) Treatment with anticancer agents (including investigational drugs) less than 2 weeks before first study treatment administration (less than 4 weeks if the anticancer agents were antibodies).
13) Prior treatment with another selective ER down-regulator (SERD) except fulvestrant for which a washout of at least 6 weeks is required prior to the first study drug administration.
14) Inadequate hematological function including neutrophils $<1.5 \times 10^9$/L; platelet count $<100 \times 10^9$/L.
15) Prothrombin time (PT): International normalized ratio (INR) >1.5 times the upper limit of normal (ULN) or within therapeutic range if receiving anticoagulation that would affect the PT/INR.
16) Inadequate renal function with serum creatinine ≥1.5 × ULN or, if between 1.0 and 1.5 × ULN with estimated glomerular filtration rate <60 mL/min/1.73 m^2 as estimated using the abbreviated Modification of Diet in Renal Disease formula.
17) Liver function: aspartate aminotransferase >3 × ULN, or alanine aminotransferase >3 × ULN. Alkaline phosphatase up to Grade 2 (2.5 to 5 × ULN) would be acceptable only if related to the presence of bone and/or liver metastases as judged by the Investigator. Total bilirubin >1.5 × ULN.
18) Patients with Gilbert disease.
19) Non-resolution of any prior treatment-related toxicity to <Grade 2, except for alopecia according to the National Cancer Institute Common Terminology Criteria for Adverse Events (NCI-CTCAE) v4.03.
20) Treatment with drugs that have the potential to inhibit UDP-glucuronosyltransferase (including but not limited to atazanavir and probenecid), and treatments that are a P-glycoprotein sensitive substrate (including but not limited to dabigatran, digoxin, and fexofenadine), and breast cancer resistance protein

sensitive substrate (rosuvastatin, sulfasalazine), less than 2 weeks before first study treatment administration or 5 elimination half-lives whichever is longest.

21) a. All study parts, treatment with strong and moderate cytochrome P450 (CYP) 3A and CYP2C8 inducers within 2 weeks before first study treatment administration or 5 elimination half-lives whichever is longest.

b. In Part A and B only: In patients with 4β-OH cholesterol assessment: Treatment with strong and moderate CYP3A inhibitors within 2 weeks before first study treatment administration or 5 elimination half-lives whichever is longest.

22) More than 1 prior advanced , cyclin-dependent kinase 4 and 6 (CDK4/6) inhibitor-based therapy.

Definition of DLTs

DLTs were defined as the occurrence of any of the following treatment-emergent adverse events (TEAEs) related to the study therapy using NCI-CTCAE (v4.03):

- Any Grade ≥3 non-hematological toxicity, except:
  - Grade 3 nausea and vomiting resolving to Grade ≤1 within 48 hours, with or without adequate antiemetic treatment
  - Grade 3 diarrhea if controlled with adequate antidiarrheal therapy and lasting less than 48 hours.
- Any Grade ≥3 hematological toxicity, except:
  - Grade 3 anemia
  - Grade 4 neutropenia <7 days
  - Grade 3 neutropenia without fever or infection
  - Grade 3 thrombocytopenia without bleeding.
- Any elevated total serum bilirubin >2 × ULN.
- Any toxicity related to study treatment resulting in omission of the study treatment for 7 days or more during Cycle 1.
- A TEAE that in the opinion of the study committee is possibly or probably study treatment-related and is of potential clinical significance such that further dose escalation would expose patients to unacceptable risk.

These TEAEs were considered as study treatment-related in the absence of evidence to the contrary and if not related to disease progression. If multiple toxicities were seen, the presence of DLTs was based on the most severe experienced toxicity.

18F-FES PET/CT imaging and image analysis

^{18}F-FES preparation and administration

^{18}F-FES was manufactured using a modified version of the published work by Knott and colleagues, 2011 [1]. Approximately 6 mCi (185 MBq) ± 20% was drawn with a mass limit for the injected ^{18}F-FES of ≤ 5 µg (≤ 17 nmol) and the total radioactivity was assayed. ^{18}F-FES was administered via intravenous injection (bolus or via an intravenous pump over 2 minutes diluted to at least 10 mL for infusion) ideally in the hand or arm contralateral to the side of the known primary breast cancer. When the injection was complete, the intravenous catheter was immediately double-flushed with at least 2 × 10 mL of saline. The dose syringe was then re-assayed for total radioactivity. If the residual activity was 0.1 mCi or greater, the recorded amount of the injected dose was corrected for residual activity.

Imaging procedures

Imaging was performed 60 ± 10 minutes after injection using an integrated PET/CT scanner. A low-dose CT scan was acquired from skull base to mid-thigh without contrast for attenuation correction and anatomical localization of lesions identified in the PET scan. After the CT scan, a 3D emission PET scan covering the same axial field of view was performed. PET data were reconstructed iteratively with segmented correction for attenuation with the CT data and displayed in multiplanar reconstructions. Reconstruction algorithms varied by imaging site but were consistent within each site and for each individual patient.

Interpretation of ^{18}F-FES PET/CT scans

One board-certified physician, experienced in ^{18}F-FES PET/CT, reviewed the ^{18}F-FES PET/CT examinations performed at their respective sites. Physiologic accumulation and excretion of ^{18}F-FES avidity was expected in the liver, bowel, kidneys, ureters, and bladder. Focal sites of ^{18}F-FES avidity that could not be attributed to physiologic avidity were considered ^{18}F-FES-avid malignancy. Up to 5 ^{18}F-FES-avid malignant lesions were chosen as index lesions on the pretreatment scans. Index lesions were chosen based on their ease of reproducible localization and ^{18}F-FES standardized uptake value (SUV) by body weight measurement, and not necessarily those with maximal uptake. Bone and nodal lesions were preferred for index lesions. Other acceptable index lesion sites included the chest wall, pleura, adrenal, and other soft

tissue masses. Liver lesions were only measured if lesions were appreciably above physiologic liver avidity and there were no other suitable index lesions, as physiologic liver avidity limits evaluation. Patients with liver metastases were allowed into the study only if, in addition to the liver lesions, they also had lesions in other locations. Lung lesions were only measured if there were no other suitable lesions as breathing-motion may artificially change SUV measurements.

A volume of interest (VOI) was drawn to encompass each index lesion and the lesion's maximum SUV (SUV_{max}) was recorded. A background VOI was drawn in assumed normal tissue and a background SUV_{max} ($SUV_{background}$) was recorded. Only one $SUV_{background}$ was recorded per scan. For example, if the patient had ^{18}F-FES-avid index lesions in the lumbar spine, an assumed normal thoracic vertebra was chosen for the background measurement. Similarly, if the patient had ^{18}F-FES-avid index lesions in the left pleura, a section of assumed normal right pleura was chosen for the background measurement. If different sites for background measurement yielded different SUV values, the background with the highest SUV_{max} was chosen. A tumor to background ratio of 1.5 or more was required for a lesion to be labeled as an index lesion. For on-treatment ^{18}F-FES PET/CT scans, the same index lesions from the pretreatment scan were used to measure the SUV.

^{18}F-FES uptake for each lesion was calculated as $SUV_{max} – SUV_{background} = SUV_{max(cor)}$. ER occupancy was calculated as the mean percentage reduction from baseline in ^{18}F-FES $SUV_{max(cor)}$ for all index lesions within each patient.

Supplementary References

1. Knott, K., Gratz, D., Hubner, S., Juttler, S., Zankl, C., & Muller, M. Simplified and automatic one-pot synthesis of 16a-[18F]fluoroestradiol without high performance liquid chromatography purification. *J. Labelled Comp. Radiopharm.* **54,**749–753 (2011).

Supplementary Table S1: Amcenestrant plasma pharmacokinetic parameters (48h pharmacokinetic profiles) following a single administration of amcenestrant (Cycle 1, Day 1) under fasting conditions during Part A dose escalation.

	Amcenestrant dose level during Part A dose escalation				
	**20 mg**	**150 mg**	**200 mg**	**400 mg**	**600 mg**
**N**	3	3	4	3	3
**t_{max}, h**					
Median	1.52	3.00	2.98	3.00	3.03
Min–Max	1.50–2.00	3.00–24.77	1.98–4.02	1.50–3.83	2.02–4.00
**C_{max}, ng/mL**					
Mean ± SD	187 ± 46.7	1310 ± 1380	1650 ± 1340	4740 ± 2920	7010 ± 4180
Geometric mean (CV%)	183 (24.9)	884 (105.3)	1340 (81.4)	4120 (61.5)	6160 (59.6)
**$AUC_{0\text{-}24h}$, ng•h/mL**					
Mean ± SD	1040 ± 560	9140 ± 8730	13600 ± 13800	40400 ± 17500	61100 ± 36300
Geometric mean (CV%)	924 (53.8)	6860 (95.6)	9760 (101.3)	37600 (43.2)	53400 (59.5)

$AUC_{0\text{-}24h}$, Area under the plasma concentration-time curve from time zero to 24 h; C_{max}, maximum (peak) plasma drug concentration; CV, coefficient of variation; SD, standard deviation; t_{max}, time to reach maximum (peak) plasma concentration following drug administration.

Supplementary Table S2: Amcenestrant plasma pharmacokinetic parameters following repeated once-daily administrations of amcenestrant (Cycle 1, Day 22) under fasting conditions during Part A dose escalation.

	**Amcenestrant dose level during Part A dose escalation**				
	**20 mg**	**150 mg**	**200 mg[a]**	**400 mg**	**600 mg**
**N**	3	2	3	3	3
**t_{max}, h**					
Median	2.17	2.97	2.07	2.95	3.00
Min–Max	2.00–4.02	2.93–3.00	2.00–3.03	2.02–3.78	3.00–4.00
**C_{max}, ng/mL**					
Mean ± SD	218 ± 95.3	2390 ± 1200	2150 ± 873	4020 ± 2460	5570 ± 962
Geometric mean (CV%)	203 (43.7)	2230 (50.1)	2030 (40.7)	3370 (61.3)	5510 (17.3)
**$AUC_{0\text{-}24}$, ng•h/mL**					
Mean ± SD	1630 ± 1120	15900 ± 7350	13900 ± 4380	36800 ± 23500	42700 ± 11200
Geometric mean (CV%)	1350 (68.8)	15000 (46.3)	13400 (31.6)	29500 (63.8)	41700 (26.2)
**CL_{ss}/F, L/h**					
Mean ± SD	18.0 ± 13.7	10.6 ± 4.89	15.3 ± 4.30	18.1 ± 17.4	14.7 ± 3.96
Geometric mean (CV%)	14.8 (75.8)	9.99 (46.3)	14.9 (28.0)	13.5 (95.7)	14.4 (26.9)
**C_{trough}, ng/mL**					
Mean ± SD	12.6 ± 13.6	135 ± 21.2	140 ± 102	397 ± 315	449 ± 396
Geometric mean (CV%)	17.0 (108.3)	134 (15.7)	115 (73.0)	245 (79.3)	350 (88.2)

[a]Profile of one patient was excluded; $AUC_{0\text{-}24h}$, Area under the plasma concentration-time curve from time zero to 24 h; CL_{ss}/F, apparent total clearance of the drug from plasma at steady state after oral administration; C_{max}, maximum (peak) plasma drug concentration; C_{trough}, trough plasma concentration (measured concentration at the end of a dosing interval at steady state [taken directly before next administration]); CV, coefficient of variation; SD, standard deviation; t_{max}, time to reach maximum (peak) plasma concentration following drug administration.

Supplementary Table S3: Accumulation for amcenestrant after QD administration – Descriptive statistics for accumulation ratio by dose level during Part A dose escalation.

	Amcenestrant dose level					
	**20 mg (N=3)**	**150 mg (N=3)**	**200 mg (N=4)**	**400 mg (N=3)**	**600 mg (N=3)**	**All (N=16)**
**$R_{ac}AUC_{0-24}$**						
Number	3	2	3	3	3	14
Geometric Mean	1.465	1.695	2.087	0.787	0.782	1.234
90% CI	0.889, 2.413	0.920, 3.125	1.266, 3.438	0.477, 1.296	0.474, 1.288	0.998, 1.594
**$R_{ac}C_{max}$**						
Number	3	2	3	3	3	14
Geometric Mean	1.107	1.569	2.111	0.818	0.894	1.196
90% CI	0.720, 1.701	0.927, 2.656	1.374, 3.244	0.532, 1.257	0.582, 1.374	0.996, 1.490

$R_{ac}AUC_{0-24}$ = accumulation ratio based upon AUC_{0-24} and is defined as the AUC_{0-24} on Cycle 1 Day 22 divided by the AUC_{0-24} on Cycle 1 Day 1.

$R_{ac}C_{max}$ = accumulation ratio based upon C_{max} and is defined as the C_{max} on Cycle 1 Day 22 divided by the C_{max} on Cycle 1 Day 1.

AUC_{0-24h}, area under the plasma concentration-time curve from time zero to 24 h; C_{max}, maximum (peak) plasma drug concentration; CI, confidence interval.

Supplementary Table S4: Dose proportionality assessment for amcenestrant (all doses) – estimates with 90% CI for r-fold increases during Part A dose escalation.

		Ratio Cycle 1 Day 1		Ratio Cycle 1 Day 22	
**Parameter**	**Dose ratio**	**Estimate**	**90% CI**	**Estimate**	**90% CI**
**$AUC_{0\text{-}24}$**	(r) = 2	2.306	1.911 to 2.783	2.014	1.735 to 2.338
	(r) = 30	60.328	23.978 to 151.753	31.059	14.939 to 64.578
	Beta Estimate	1.205	0.934 to 1.477	1.010	0.795 to 1.225
**C_{max}**	(r) = 2	2.049	1.707 to 2.460	1.938	1.701 to 2.209
	(r) = 30	33.770	13.774 to 82.793	25.734	13.564 to 48.823
	Beta Estimate	1.035	0.771 to 1.299	0.955	0.767 to 1.143

$AUC_{0\text{-}24h}$, area under the plasma concentration-time curve from time zero to 24 h; CI, confidence interval; C_{max}, maximum (peak) plasma drug concentration.

Supplementary Table S5: Food effect on AUC_{0-24} and C_{max} following a single oral administration from 20 mg to 600 mg of amcenestrant - Estimate and 90% CI for the ratio of food condition geometric means (fed/fasted) during Part A dose escalation.

	Number of participants included in the model	Comparison	Estimate	90% CI
AUC_{0-24}	2	Fed versus Fasted (20 mg)	1.05	(0.60 to 1.83)
	1	Fed versus Fasted (150 mg)	0.69	(0.31 to 1.50)
	4	Fed versus Fasted (200 mg)	1.77	(1.20 to 2.62)
	1	Fed versus Fasted (400 mg)	1.12	(0.51 to 2.45)
	3	Fed versus Fasted (600 mg)	1.38	(0.88 to 2.17)
C_{max}	2	Fed versus Fasted (20 mg)	0.80	(0.41 to 1.58)
	1	Fed versus Fasted (150 mg)	0.41	(0.16 to 1.07)
	4	Fed versus Fasted (200 mg)	1.67	(1.04 to 2.69)
	1	Fed versus Fasted (400 mg)	1.38	(0.53 to 3.59)
	3	Fed versus Fasted (600 mg)	1.43	(0.83 to 2.48)

AUC_{0-24h}, area under the plasma concentration-time curve from time zero to 24 h; CI, confidence interval; C_{max}, maximum (peak) plasma drug concentration.

Supplementary Table S6: 4β-hydroxycholesterol posttreatment/pretreatment ratio at 4 weeks after the start of amcenestrant dosing.

	Part A					Part B
**Amcenestrant dose**	**20 mg (N=3)**	**150 mg (N=3)**	**200 mg (N=4)**	**400 mg (N=3)**	**600 mg (N=3)**	**400 mg (N=49)**
**Number**	3	3	4	3	2	12
**Mean (SD)**	1.09 (0.304)	1.13 (0.432)	1.39 (0.207)	1.83 (0.569)	2.40 (0.559)	1.89 (0.627)
**Median**	0.953	1.28	1.34	1.69	2.40	1.77
**Geometric Mean**	1.06	1.06	1.37	1.78	2.37	1.80
**95% CI**	(0.332, 1.842)	(0.057, 2.203)	(1.057, 1.715)	(0.419, 3.247)	(−2.622, 7.418)	(1.491, 2.288)
**Min ; Max**	0.873 ; 1.43	0.644 ; 1.47	1.20 ; 1.66	1.35 ; 2.46	2.00 ; 2.79	1.08 ; 3.14
**CV**	28.0	38.2	14.9	31.1	23.3	33.2

CI, confidence interval; CV, coefficient of variation; SD, standard deviation.

Supplementary Table S7. Listing of serious treatment-emergent adverse events occurring during Part B dose expansion.

Patient	Preferred term
1	Disease progression<br>Death due to pneumonia
2	Dysphagia<br>Dyspnea<br>Pulmonary embolism
3	Jaundice, cholestatic
4	Spinal pain
5	Hypercalcemia
6	Asthenia<br>Dyspnea<br>Pyrexia
7	Cancer pain
8	Death due to unknown cause
9	Hyperbilirubinemia
10	Nausea<br>Tumor pain<br>Vomiting
11	Cellulitis
12	Breast cellulitis
13	Pulmonary embolism

Supplementary Table S8: Amcenestrant plasma pharmacokinetic parameters following a single administration of amcenestrant (Cycle 1, Day1) and repeated once-daily administrations of amcenestrant (Cycle 1, Day 22) during Part B dose expansion.

	**Amcenestrant 400 mg QD during Part B dose expansion**	
	**Day1**	**Day 22**
**N**	16	13
**T_{lag}, h**		
Median	0	NA
Min–Max	0–1.0	NA
**t_{max}, h**		
Median	3.98	3.00
Min–Max	1.97–9.98	1.02–6.00
**C_{max}, ng/mL**		
Mean ± SD	6020 ± 2810	4380 ± 1230
Geometric mean (CV%)	5460 (46.8)	4220 (28.0)
**AUC_{0-24}, ng•h/mL**		
Mean ± SD	50900 ± 26000	43200 ± 16200
Geometric mean (CV%)	44800 (51.1)	40400 (37.5)
**CL_{ss}/F, L/h**		
Mean ± SD	NA	10.6 ± 4.35
Geometric mean (CV%)	NA	9.90 (41.0)
**C_{trough}, ng/mL**		
Mean ± SD	NA	630 ± 550
Geometric mean (CV%)	NA	466 (87.3)

AUC_{0-24h}, Area under the plasma concentration-time curve from time zero to 24 h; CL_{ss}/F, apparent total clearance of the drug from plasma at steady state after oral administration; C_{max}, maximum (peak) plasma drug concentration; C_{trough}, trough plasma concentration (measured concentration at the end of a dosing interval at steady state [taken directly before next administration]); CV, coefficient of variation; NA, not applicable; SD, standard deviation; t_{lag}, lag time; t_{max}, time to reach maximum (peak) plasma concentration following drug administration.

Supplementary Table S9. List of study investigators, sites, settings, and approval centers

Country	Investigator name	Site name	Town	Setting	IRB/IEC
Belgium	Patrick NEVEN	UZ	Leuven	University hospital	National: Ethische Commissie Onderzoek UZ/KU, Leuven Herestraat 49, Leuven 3000, BELGIUM
Canada	Ellen WARNER	Sunnybrook Research Institute	Toronto	Research and teaching hospital	Local: Sunnybrook Health Sciences Centre Research Ethics Office, Room C819, 2075 Bayview Avenue, Toronto, M4N 3M5 Ontario, CANADA
Czech Republic	Martina ZIMOVJANOVA	Vseobecna fakultni nemocnice	Praha	Research and teaching hospital	Local: Eticka komise Vseobecne FN v Praze, Na Bojisti 1, Praha 2, 12808, CZECH REPUBLIC<br><br>National: EK při IKEM a Thomayerově nemocnici, Videnska 800, Praha 4 - Krc 14059, CZECH REPUBLIC
Czech Republic	Katarina PETRAKOVA	Masarykuv onkologicky ustav	Brno	Cancer treatment and research center	Local: Eticka komise Masarykuv onkologicky ustav, Zluty kopec 7, Brno, 65653, CZECH REPUBLIC<br><br>National: EK při IKEM a Thomayerově nemocnici, Videnska 800, Praha 4 – Krc, 14059, CZECH REPUBLIC
Czech Republic	Tomas BUCHLER	Fakultni Thomayerova nemocnice	Praha	University hospital	Local: EK při IKEM a Thomayerově nemocnici, Videnska 800, Praha 4 – Krc 14059, CZECH REPUBLIC<br><br>National: EK při IKEM a Thomayerově nemocnici, Videnska 800, Praha 4 – Krc, 14059, CZECH REPUBLIC
France	Mario CAMPONE	Site René Gauducheau	Nantes	Cancer treatment and research center	National: CPP Sud-Méditerranée 4, Centre Hôspitalier Universitaire de Montpellier, Hôpital Saint Eloi, 2, avenue Bertin Sans, Montpellier Cedex 05 34295, FRANCE
France	Thomas BACHELOT	Centre Léon Bérard	Lyon	Research and teaching hospital	National: CPP Sud-Méditerranée 4, Centre Hôspitalier Universitaire de Montpellier, Hôpital Saint Eloi, 2, avenue Bertin Sans, Montpellier Cedex 05 34295, FRANCE
France	Nawale HAJJAJI	Centre Oscar Lambret	Lille	Cancer treatment and research center	National: CPP Sud-Méditerranée 4, Centre Hôspitalier Universitaire de Montpellier, Hôpital Saint Eloi, 2, avenue Bertin Sans, Montpellier Cedex 05 34295, FRANCE
Portugal	Sofia BRAGA	CUF Descobertas	Lisbon	Hospital	National: CEIC - Comissão de Ética para a Investigação Clínica, Parque da Saúde de Lisboa, Pav. 17-A Avenida do Brasil nº 53, Lisboa, 1749-004, PORTUGAL
Spain	Eva CIRUELOS GIL	Hospital 12 de Octubre	Madrid	University hospital	National: CEIM HM Hospitales Avenida de Montepríncipe 25 Boadilla Del Monte, 28660 Madrid, SPAIN
Spain	Valentina BONI	Centro Integral Oncológico Clara Campal	Madrid	Cancer treatment and research center	National: CEIM HM Hospitales Avenida de Montepríncipe 25 Boadilla Del Monte, 28660 Madrid, SPAIN

UK	Simon LORD	Churchill Hospital	Oxford	Teaching hospital	National: West Midlands Coventry & Warwick Research Ethics Committee, The Old Chapel, Royal Standard Place, Nottingham, NG1 6FS, Nottinghamshire, UNITED KINGDOM
US	Hannah LINDEN	Seattle Cancer Care Alliance	Seattle	Cancer treatment and research center	Local: Western Institutional Review Board, 1019 39th Avenue SE, Suite 120, Puyallup, 98374-2115 Washington, UNITED STATES
US	Aditya BARDIA	Massachusetts General Hospital Cancer Center	Boston	Research hospital	Local: Dana Farber, 450 Brookline Ave OS229, Boston, 02215 Massachusetts, UNITED STATES
US	Sarat CHANDARLAPATY	Memorial Sloan Kettering Cancer Center	New York	Cancer treatment and research center	Local: Institutional Review Board, Memorial Sloan-Kettering Cancer, 1275 York Avenue, New York, 10065 New York, UNITED STATES
US	Peter KABOS	University of Colorado	Aurora	University hospital	Local: Western Institutional Review Board, 1019 39th Avenue SE, Suite 120, Puyallup, 98374-2115 Washington, UNITED STATES

IRB, Institutional Review Board; IEC, Independent Ethics Committee.
