# Supplementary material for: AMEERA-1 phase 1/2 study of amcenestrant, SAR439859, in postmenopausal women with ER-positive/HER2-negative advanced breast cancer
Source: Nat Commun. 2022 Jul 15;13:4116. doi: 10.1038/s41467-022-31668-8 (PMC9284491; doi:10.1038/s41467-022-31668-8)
Supplement: Supplementary file 2 — Reporting Summary [file 41467_2022_31668_MOESM2_ESM.pdf]

nature research

Corresponding author(s): Mario Campone

Last updated by author(s): Apr 8, 2022

Reporting Summary

Nature Research wishes to improve the reproducibility of the work that we publish. This form provides structure for consistency and transparency in reporting. For further information on Nature Research policies, see our Editorial Policies and the Editorial Policy Checklist.

Statistics

For all statistical analyses, confirm that the following items are present in the figure legend, table legend, main text, or Methods section.

n/a	Confirmed	
☐	☒	The exact sample size (n) for each experimental group/condition, given as a discrete number and unit of measurement
☐	☒	A statement on whether measurements were taken from distinct samples or whether the same sample was measured repeatedly
☐	☒	The statistical test(s) used AND whether they are one- or two-sided<br>*Only common tests should be described solely by name; describe more complex techniques in the Methods section.*
☒	☐	A description of all covariates tested
☒	☐	A description of any assumptions or corrections, such as tests of normality and adjustment for multiple comparisons
☐	☒	A full description of the statistical parameters including central tendency (e.g. means) or other basic estimates (e.g. regression coefficient) AND variation (e.g. standard deviation) or associated estimates of uncertainty (e.g. confidence intervals)
☒	☐	For null hypothesis testing, the test statistic (e.g. F, t, r) with confidence intervals, effect sizes, degrees of freedom and P value noted<br>*Give P values as exact values whenever suitable.*
☒	☐	For Bayesian analysis, information on the choice of priors and Markov chain Monte Carlo settings
☒	☐	For hierarchical and complex designs, identification of the appropriate level for tests and full reporting of outcomes
☒	☐	Estimates of effect sizes (e.g. Cohen's d, Pearson's r), indicating how they were calculated

Our web collection on statistics for biologists contains articles on many of the points above.

Software and code

Policy information about availability of computer code

Data collection	Clinical data was collected in electronic case report forms using Medidata Rave v2020.3.2. RNA sequencing data was collected as Fastq files.
Data analysis	Non-compartmental pharmacokinetic analysis was performed with Pheonix software (v2). FES-PET image analysis was processed with Pmod (v3.905)<br>For RNA sequencing and ER activity scores, the following softwares were used:<br>• STAR (v2.4.0h1)<br>• Cufflink (v2.2.1)<br>• RNA-SeQC (v1.1.7)<br>• oppar (v1.16.0)<br>• GSVA (v1.36.3)<br>• GSEABase (v1.50.1)<br>Statistical analysis was performed with SAS v9.4 and R software v3.5.3.<br>Figures were created in GraphPad Prism version 7.05.

For manuscripts utilizing custom algorithms or software that are central to the research but not yet described in published literature, software must be made available to editors and reviewers. We strongly encourage code deposition in a community repository (e.g. GitHub). See the Nature Research guidelines for submitting code & software for further information.

Data

Policy information about availability of data

All manuscripts must include a data availability statement. This statement should provide the following information, where applicable:

- Accession codes, unique identifiers, or web links for publicly available datasets
- A list of figures that have associated raw data
- A description of any restrictions on data availability

Qualified researchers may request access to patient level data and related study documents including the clinical study report, study protocol with any amendments, blank case report form, statistical analysis plan, and dataset specifications for legitimate research purposes, as evaluated by an independent scientific review board. Patient level data will be anonymized and study documents will be redacted to protect the privacy of our trial participants. Access to data may be restricted in cases where data collected is subject to contractual or consent provisions that prohibit transfer to third parties. Data use agreements will be used for approved data requests. Access to data is expected to take a few months, and will depend on the number of data contributors, the number of studies, and the requestor's availability to respond to comments. To submit a request and for further details on Sanofi's data sharing criteria, eligible studies, and process for requesting access can be found at: https://www.vivli.org/.

Field-specific reporting

Please select the one below that is the best fit for your research. If you are not sure, read the appropriate sections before making your selection.

☒ Life sciences ☐ Behavioural & social sciences ☐ Ecological, evolutionary & environmental sciences

For a reference copy of the document with all sections, see nature.com/documents/nr-reporting-summary-flat.pdf

Life sciences study design

All studies must disclose on these points even when the disclosure is negative.

Sample size	A minimax Simon 2-stage design with 5% 1-sided Type 1 error rate was used to test the null hypothesis of a 10% response rate. Under the alternative assumption of 20% response rate, a total of 78 patients were needed to guarantee at least 80% power, accounting for the binding futility interim analysis. In the first stage 45 patients were accrued. If 4 responders or fewer by ICR in these 45 patients were observed, Part B would be stopped. The null hypothesis would be rejected if ≥13 responders were observed in the 78 patients.
Data exclusions	Various analysis populations were employed in the study with rationales as follows.<br>The DLT-evaluable population during Part A planned to include all patients who had received a first complete cycle, who had received at least 75% of the intended dosing (unless the patient discontinued treatment before Cycle 1 completion due to a DLT) and had evaluable 18F-FES PET/CT scans at baseline and between Day 11 and Day 15 of the first cycle. Results were analyzed using descriptive statistics.<br>The safety population included all patients exposed to at least one dose of the study treatment.<br>The response-evaluable population was defined as treated patients with measurable disease at study entry who had at least one post-baseline evaluable tumor assessment. Patients with an early progression as per RECIST v1.1 or who died from disease progression were evaluable for response.<br>The pharmacokinetic-population evaluable for non-compartmental pharmacokinetic analysis (NCA) was defined as all patients from the all-treated population without any major deviations related to study treatment administration (e.g., early vomiting just after drug administration, food status), and who had adequate blood samples enabling determination of at least one pharmacokinetic parameter.<br>The biomarker-evaluable population was defined as all patients with available biomarker data from the response-evaluable pooled population of patients from Parts A and B who had received an amcenestrant dose ≥150 mg excluding the pharmacodynamically inactive 20-mg dose. Only paired samples (screening or Cycle 1, Day1 and available Cycle 2, Day 28 samples) were considered for assessment of the evolution of biomarkers over time.
Replication	Replication was not applicable to this study as this was a clinical study with unique patient samples. However, for the assessment of best tumor responses as per RECIST v1.1, such as complete response (CR) or partial response (PR), all tumor responses had to be confirmed on a second examination done at least 4 weeks apart, in order to validate the antitumoral response. We affirm that all tumor responses reported in the manuscript were confirmed (replicated) responses.
Randomization	This was a first-in-human, Phase 1/2, open label, single-arm study, whose design is standard in oncology. As such, randomization is not relevant here.
Blinding	This was a first-in-human, Phase 1/2, open label, single-arm study, whose design is standard in oncology. As such, blinding is not relevant here.

Reporting for specific materials, systems and methods

We require information from authors about some types of materials, experimental systems and methods used in many studies. Here, indicate whether each material, system or method listed is relevant to your study. If you are not sure if a list item applies to your research, read the appropriate section before selecting a response.

Materials & experimental systems

n/a	Involved in the study	
	x	Antibodies
x		Eukaryotic cell lines
x		Palaeontology and archaeology
x		Animals and other organisms
	x	Human research participants
	x	Clinical data
x		Dual use research of concern

Methods

n/a	Involved in the study	
x		ChIP-seq
x		Flow cytometry
x		MRI-based neuroimaging

Antibodies

Antibodies used	Immunohistochemistry staining of formalin-fixed paraffin-embedded (FFPE) tumor tissue sections was performed on the Ventana Discovery XT IHC platform using anti-ER clone SP1 (CONFIRM Anti-Estrogen Receptor, Roche, ref. 790-4325), anti-PgR clone 1E2 (CONFIRM Anti-progesterone receptor, Roche, ref. 790-4296), and anti-Ki67 clone 30-9 (CONFIRM Anti-Ki-67, Roche, Ref. 790-4286).
Validation	Each FFPE tumor issue section was run with the primary antibody (ER, PgR, and Ki67) and a negative control reagent (isotypic control; CONFIRM negative control rabbit Ig antibody). Commercially procured FFPE positive and negative tissue controls were also included in each run as described below:<br>• Human endometrium samples were used as positive and negative samples for ER and PgR staining; positive staining is expected in epithelial glandular, stromal and smooth muscle cells, but there are known cellular components (cytoplasm, membrane) and tissue components (blood vessels) that are not stained with ER or PgR.<br>• Normal human colon was used as positive and negative samples for Ki67 staining; positive staining was expected in colonic crypts, but there are known cellular components (cytoplasm, membrane) and tissue components (muscle) that are not stained with Ki67.<br>A set of two slides with appropriate control tissue for each biomarker was included in each run; one slide was incubated with the primary antibody and the other with the isotypic control antibody.<br>• For ER staining, per CONFIRM Anti-Estrogen Receptor, Roche, ref. 790-4325, for the test to be considered valid, the positive control tissue should exhibit nuclear staining of the tumor cells or uterine glands and stroma. These components should be negative when stained with CONFIRM Negative Control Rabbit Ig. In addition, it is recommended that a negative tissue control slide (for example, an ER negative breast carcinoma) be included for every batch of samples processed and run on a BenchMark IHC/ISH instrument.<br>Certificate of Analysis documents provided on manufacturer's website require passing of the following criteria on Breast Carcinoma Tissue: Nuclear staining is interpreted on tissue that is positive for percent tumor cell staining according to the following approximate percent tumor cell staining criteria: 0-50% cell staining (negative) 51-100% cell staining (positive). Nuclear staining of test and reference slides must score more than or equal 3.5 for specific staining with a background (non-specific) intensity of less than or equal 0.5. The specific staining intensity of the test must be within a 0.5 intensity score of the reference slide for a passing result (Scale 0-4). The intensity of the negative reagent control slide must be 0.5 or less (Scale 0-4).<br>• For PR staining, per CONFIRM Anti-Progesterone Receptor, Roche, ref. 790-4296, for the test to be considered valid, the positive control tissue should exhibit nuclear staining of the tumor cells or uterine glands and stroma. These components should be negative when stained with CONFIRM Negative Control Rabbit Ig. In addition, it is recommended that a negative tissue control slide (for example, a PR negative breast carcinoma) be included for every batch of samples processed and run on the BenchMark IHC/ISH instrument. This negative tissue control should be stained with CONFIRM anti-PR (1E2) antibody to ensure that the antigen enhancement and other pretreatment procedures did not create false positive staining.<br>Certificate of Analysis documents provided on manufacturer's website require passing of the following criteria on Breast Carcinoma Tissue: Nuclear staining is interpreted on tissue that is positive for percent tumor cell staining according to the following approximate percent tumor cell staining criteria: 0-50% cell staining (negative) 51-100% cell staining (positive). Nuclear staining of test and reference slides must score #3.5 for specific staining with a background (non-specific) intensity of #0.5. The specific staining intensity of the test must be within a 0.5 intensity score of the reference slide for a passing result (Scale 0-4). The intensity of the negative reagent control slide must be 0.5 or less (Scale 0-4).<br>• For Ki-67 staining, per CONFIRM Anti-Ki-67, Roche, Ref. 790-4286, in addition to staining with CONFIRM anti-Ki-67 (30-9) antibody, a second slide should be stained with the appropriate negative control reagent. A positive tissue control must be run with every staining procedure performed. Control tissue may contain both positive and negative staining elements and serve as both the positive and negative control. If the positive tissue controls fail to demonstrate positive staining, results of the test specimen should be considered invalid.<br>Certificate of Analysis documents provided on manufacturer's website require passing of the following criteria on breast carcinoma, normal tonsil, or normal lymph node: The test batch must stain 51% or greater of cells on breast carcinoma, normal tonsil, or normal lymph node tissue. The specific stain intensity must also be within 0.5 pt. of the reference batch for a passing result. The intensity of the negative reagent control slide must be 0.5 or less.

Human research participants

Policy information about studies involving human research participants

Population characteristics	Postmenopausal women with ER+/HER2– breast cancer, with measurable disease by RECIST v1.1, who had received ≥6 months prior endocrine therapy, and an ECOG score of 0 or 1. Patients were excluded from the study if they had known brain metastases, leptomeningeal carcinomatosis and/or spinal cord compression, prior SERD treatment except fulvestrant with a ≥6-week washout period.
Recruitment	Patients were recruited from 16 sites in 8 countries according to strict predefined inclusion and exclusion criteria as specified in the protocol.<br>Recruitment was facilitated by public disclosure of the trial on clinicaltrials.gov. Patients were also recruited by the clinical investigator within their clinic by identifying eligible patients using protocol-specified inclusion and exclusion criteria. Eligible patients were offered participation in the clinical trial and had the opportunity to accept or decline. Since patients are recruited from the hospital site in a particular city, this may lead to bias by catering to a specific geographic area. However, this is mitigated by the participation of multiple global clinical sites in this study. The inclusion/exclusion criteria may also select for a specific subgroup of patients and therefore results in the wider population may differ from the results of the trial. Real world evidence studies are thus important to conduct to confirm the applicability of the results to a wider patient population.
Ethics oversight	The protocol was approved by the institutional review board or independent ethics committee at each site (see Supplementary Table S9 for the names of the boards/committees who provided approval) and complied with the International Ethical Guidelines for Biomedical Research Involving Human Subjects, Good Clinical Practice guidelines, the Declaration of Helsinki, and local laws. All patients provided written informed consent and no compensation was offered.

Note that full information on the approval of the study protocol must also be provided in the manuscript.

Clinical data

Policy information about clinical studies

All manuscripts should comply with the ICMJEguidelines for publication of clinical research and a completedCONSORT checklist must be included with all submissions.

Clinical trial registration	NCT03284957
Study protocol	The study protocol is provided in the additional review material accompanying this article
Data collection	Patients were recruited from 16 sites in 8 countries and in a variety of settings as per Supplementary Table S10. The first patient was enrolled on Nov 6, 2017 and the last patient was enrolled on March 26, 2020. Each patient had data collected until the end of treatment, which was 22–30 days after the last administration of study treatment.
Outcomes	The primary outcomes of this study were dose-limiting toxicities, and determination of the maximum tolerated dose and recommended Phase 2 dose in Part A, and the ORR in Part B. Secondary outcomes included:<br>• Safety<br>• PK<br>• ORR and CBR by RECIST v1.1<br>• Time to first tumor response<br>• ER availability<br>Exploratory outcomes included:<br>• PK/PD relationships<br>• Gene mutational profiles<br>• Breast cancer biomarkers
